# From Xenobiotic Exposure to Neuroinflammation: Mechanisms Linking Lipopolysaccharide Signaling to Depressive-like Behavior

**DOI:** 10.3390/jox16040129

**Published:** 2026-07-10

**Authors:** Alissa Maria de Oliveira Martins, Maxsyara Felismino da Silva Soares, Lucas Nóbrega de Oliveira, Nayana M. M. V. Barbosa, André Luiz Leocádio de Souza Matos, Maria Clara Ferreira Gonçalves, Adriana M. F. de Oliveira-Golzio, Cícero Francisco Bezerra Felipe, Marcus T. Scotti, Pablo R. da Silva, Luciana Scotti

**Affiliations:** 1 Psychopharmacology Laboratory, Institute for Drug and Medicine Research, Federal University of Paraíba, João Pessoa 58051-900, PB, Brazilnayanamedeiros@ltf.ufpb.br (N.M.M.V.B.); andre.leocadio@academico.ufpb.br (A.L.L.d.S.M.);; 2 Postgraduate Program in Natural and Synthetic Bioactive Products, Health Sciences Center, Federal University of Paraíba, João Pessoa 58051-900, PB, Brazil; 3 Postgraduate Program in Cognitive and Behavior Neuroscience, Center for Humanities, Literature and Arts, Federal University of Paraíba, João Pessoa 58051-900, PB, Brazil; 4 Department of Molecular Biology, Center for Exact and Natural Sciences, Federal University of Paraíba, João Pessoa 58051-900, PB, Brazil; 5 Chemoinformatics Laboratory, Institute for Drug and Medicine Research, Federal University of Paraíba, João Pessoa 58051-900, PB, Brazil; mtscotti@gmail.com

**Keywords:** lipopolysaccharide, neuroinflammation, NLRP3 inflammasome, oxidative stress, mitochondrial dysfunction, depression-like behavior, microglia, kynurenine pathway, xenobiotics, neuroimmune signaling

## Abstract

Depression is increasingly recognized as a multifactorial disorder involving immune, metabolic, and neurobiological disturbances that extend beyond classical monoaminergic hypotheses. Among xenobiotic-based experimental approaches, lipopolysaccharide (LPS) has become a valuable tool for investigating how peripheral inflammatory stimuli are translated into central neurobiological dysfunction. This narrative review aimed to integrate current evidence regarding the mechanisms by which LPS-induced neuroimmune activation contributes to depression-related phenotypes and to discuss the translational relevance of these findings. Literature searches were performed in PubMed, ScienceDirect, and Google Scholar, focusing on studies addressing inflammatory signaling, oxidative imbalance, inflammasome activation, neurotransmitter dysfunction, and experimental modeling strategies. Current evidence suggests that LPS-induced neuroinflammation involves a dynamic interaction between peripheral immune signaling, mitochondrial dysfunction, redox imbalance, and glial activation, establishing self-amplifying mechanisms capable of sustaining chronic inflammatory states. Such alterations profoundly affect kynurenine metabolism, glutamatergic homeostasis, and reward-related neurotransmission, thereby contributing to synaptic dysfunction and behavioral impairment. Experimental findings obtained from animal, cellular, and computational platforms further support the emergence of integrated therapeutic strategies targeting multiple neuroimmune pathways. Collectively, these observations reinforce the concept that neuroinflammation represents a central biological interface linking xenobiotic exposure to depressive-like behavior.

## 1. Introduction

Depression is a mental disorder characterized by depressed mood, loss of pleasure, and reduced interest in performing daily activities. This disorder can affect all aspects of an individual’s life, including relationships with people around them, which may culminate in problems at school or work, therefore constituting a disorder with disabling potential. It is estimated that 4% of the global population suffers from depression, which, by extrapolation, corresponds to approximately 332 million individuals, being more common in women than in men [1].

Depressive disorder is a multifactorial condition in which social, psychological, genetic, and neurobiological factors contribute to its development, extending beyond the previously established monoaminergic hypothesis [2]. Evidence accumulated over recent decades has positioned inflammation as a central biological factor in the onset and progression of depressive symptoms [3,4]. This occurs because the activation of immune cells residing in the brain, especially microglia, promotes the release of inflammatory mediators, reactive oxygen species (ROS), and nitric oxide, contributing to synaptic dysfunction, impaired neuroplasticity, and neuronal damage [5]. In this context, the possibility that exogenous agents may trigger neuroimmunological responses has received increasing attention [6]. Clinical evidence also corroborates the association between inflammation and depression, as elevated circulating levels of inflammatory biomarkers, including C-reactive protein (CRP), interleukin-6 (IL-6), and tumor necrosis factor-alpha (TNF-α), have been associated with reduced antidepressant responsiveness and, consequently, a greater likelihood of treatment-resistant depression [7,8,9,10].

To investigate the causal relationship between inflammation and depressive behavior, experimental models using lipopolysaccharide (LPS) have been widely employed [11,12]. LPS is a component of the outer membrane of Gram-negative bacteria; therefore, being a substance extrinsic to the metabolism of a living organism, this endotoxin is classified as a xenobiotic [13,14,15]. LPS acts as a potent inducer of the innate immune response through activation of Toll-like receptor 4 (TLR4). Systemic administration of this substance in animal models reproduces several behavioral and biochemical characteristics observed in depression, including reduced locomotor activity [16] as a behavioral sign, and increased production of pro-inflammatory cytokines, as well as activation of inflammatory signaling pathways, such as nuclear factor kappa B (NF-κB) and mitogen-activated protein kinases (MAPKs), as neurochemical markers [17]. These models have been fundamental for elucidating how peripheral inflammatory stimuli are translated into central neurobiological alterations.

Following peripheral administration, LPS activates innate immune pathways predominantly through Toll-like receptor 4 (TLR4), particularly in macrophages, monocytes, dendritic cells, and endothelial cells, initiating systemic inflammatory signaling and triggering a cascade of inflammatory events that includes microglial activation, cytokine release, oxidative stress, NLRP3 inflammasome signaling, and alterations in neurotransmitter signaling [17]. These inflammatory events contribute to the functional imbalance of the central nervous system, making LPS, therefore, a versatile model to mimic several disorders that have neuroinflammation as a pathophysiological basis. However, considering the emphasis given here, these responses collectively contribute to behavioral alterations that resemble depressive phenotypes and provide a valuable framework for understanding inflammation-induced depression.

This review aims to integrate multiple mechanisms triggered by LPS-induced exogenous inflammatory stimulation, ranging from peripheral immune system activation to the establishment of neurobiological alterations associated with depression-like behaviors. Instead of discussing isolated pathways, this review presents an integrated view of the neuroimmune networks involved, highlighting communication between the periphery and the central nervous system, activation of TLR4/NF-κB signaling, modulation of microglial responses, NLRP3-mediated inflammatory amplification, the role of oxidative stress and mitochondrial dysfunction, as well as their consequences on neurotransmitter systems related to depression. Furthermore, emerging therapeutic strategies aimed at modulating these pathways are discussed, including anti-inflammatory, antioxidant, and inflammasome-regulating approaches, contributing to the perspective of more integrative and targeted therapies for depressive disorders associated with neuroimmune dysfunction.

## 2. Materials and Methods

In the present study, a comprehensive narrative review of the scientific literature was conducted with the aim of integrating evidence on the mechanisms linking xenobiotic exposure—particularly lipopolysaccharide (LPS)—to neuroinflammation and the development of depressive-like behavior. Literature searches were performed across widely recognized databases, including PubMed, ScienceDirect, and Google Scholar.

The search strategy employed combinations of relevant keywords and descriptors, including “lipopolysaccharide” OR “LPS”, “neuroinflammation”, “depression-like behavior”, “microglia”, “microglial activation”, “TLR4 signaling”, “NF-κB”, “NLRP3 inflammasome”, “oxidative stress”, “reactive oxygen species”, “mitochondrial dysfunction”, “blood–brain barrier”, “cytokines”, “neuroimmune signaling”, “kynurenine pathway”, “glutamatergic dysfunction”, “dopaminergic signaling”, “serotonergic system”, “xenobiotics”, and “neuroimmune pharmacology”, combined using Boolean operators (“AND” and “OR”). Priority was given to articles published in recent years, while seminal studies essential for understanding fundamental mechanisms were also included.

The literature search was conducted between March and May 2026 and included studies published up to May 2026. Priority was given to recent publications (primarily from the last 10 years), while seminal articles were retained when essential for understanding fundamental biological mechanisms. The inclusion criteria comprised peer-reviewed articles published in English, including original experimental studies (in vivo and in vitro), clinical studies, systematic reviews, meta-analyses, and narrative reviews addressing LPS-induced neuroinflammation, neuroimmune signaling, oxidative stress, neurotransmitter dysfunction, xenobiotic exposure, and depression-like behavior. Studies were required to provide mechanistic or translational evidence relevant to the scope of this review. The exclusion criteria included conference abstracts, editorials, letters to the editor, dissertations, theses, book chapters, duplicate publications, non-peer-reviewed literature, and studies not directly related to the objectives of this review. Articles lacking sufficient methodological detail or offering limited mechanistic relevance were also excluded. The literature selection strategy was based on scientific relevance, methodological quality, and conceptual contribution rather than a formal systematic review protocol. The retrieved studies were independently screened according to their titles, abstracts, and full texts when necessary. When multiple publications presented overlapping evidence, preference was given to the most comprehensive, methodologically robust, or recent studies. Particular emphasis was placed on experimental evidence elucidating the molecular and cellular mechanisms underlying LPS-induced neuroinflammation and depression-like behavior, while clinical studies and high-quality reviews were included to strengthen the translational perspective.

The selected studies were critically analyzed based on their methodological rigor and conceptual contributions to the following thematic axes: (i) mechanisms of communication between peripheral inflammatory responses and the central nervous system; (ii) microglial activation and associated signaling pathways, with emphasis on the TLR4/MyD88/TRIF/NF-κB; (iii) oxidative and nitrosative stress mechanisms induced by LPS, including NADPH oxidase activation, reactive oxygen and nitrogen species production, mitochondrial dysfunction, peroxynitrite formation, antioxidant defense impairment, and Nrf2 pathway dysregulation; (iv) the impact of neuroinflammation and oxidative stress on neurotransmitter systems, including serotonergic pathways (IDO/kynurenine pathway), glutamatergic signaling (NMDAR-mediated excitotoxicity), and dopaminergic dysfunction.

Additionally, studies employing in vivo, in vitro, and in silico experimental models were included to investigate LPS-induced neuroinflammation and the effects of xenobiotics on the central nervous system. Experimental variables such as dose, administration regimen, animal species, and route of administration were considered, as well as translational limitations associated with interspecies differences and with the inherent inability of LPS-based models to fully reproduce the heterogeneity, chronicity, psychosocial dimensions, and clinical complexity of human major depressive disorder.

The analysis also encompassed contemporary approaches to drug development, including multi-target strategies, pharmacokinetic/pharmacodynamic (PK/PD) integration, and the use of inflammatory biomarkers, with the aim of discussing translational implications for the treatment of inflammation-associated depressive disorders.

Artificial intelligence (AI)-based tools were used as complementary resources during manuscript preparation. Consensus 2.0 (San Francisco, CA, USA)was used as a supportive tool to help identify relevant scientific literature, and Rayyan (Doha, Qatar) was used to screen records and identify duplicate articles during the selection process. AI was not used to generate scientific interpretations, perform independent data analysis, or replace the authors’ critical assessment of the literature. Literature identification was conducted through conventional database searches, while AI-assisted support was limited to organizational and screening aspects. All retrieved information and references were manually verified by the authors through examination of the original publications to ensure accuracy and scientific consistency.

## 3. Neuroinflammation as a Mechanistic Link Between Peripheral Immune Activation and Depression

Communication between the peripheral immune system and the central nervous system (CNS) is a dynamic and highly regulated process that is essential for maintaining homeostasis. Under inflammatory conditions, this interaction becomes particularly relevant, allowing immune signals originating from the periphery to influence brain function and behavior. The central nervous system contains resident immune cells, particularly microglia, together with astrocytes, neurons, and other cellular components that actively participate in neuroimmune communication [18]. Commonly, a distinction is made between the central and peripheral immune systems due to the presence of the blood–brain barrier, which acts as an obstacle to the passage of immune cells originating from the periphery, as well as their mediators [19].

However, the importance of the peripheral immune component in the establishment of a neuroinflammatory state has been well documented. After peripheral exposure to inflammatory stimuli, such as LPS, peripheral immune cells contribute to the progression of neuroinflammation through the release of cytokines, such as TNF-α, IL-6, and IL-1β, which are capable of crossing the BBB and promoting microglial activation [20]. Furthermore, these cytokines can induce changes in the permeability of the blood–brain barrier, facilitating the infiltration of inflammatory mediators and peripheral immune cells into the brain parenchyma, as well as the possibility of xenobiotics, such as LPS, crossing this barrier. This barrier dysfunction, frequently observed in chronic inflammatory states, contributes to a feed-forward loop that sustains and amplifies neuroinflammation [21].

Another pathway of communication between the peripheral immune system and central immune activation occurs through neuronal communication mediated by vagal afferent fibers, which are stimulated by cytokines and pathogen-associated molecular patterns (PAMPs), and transmit this information to the nucleus tractus solitarius (NTS) in the brainstem, a key integrative center for visceral and immune-related signals. Activation of the NTS promotes downstream communication with hypothalamic and brainstem regions involved in neuroendocrine and autonomic regulation, including activation of stress-responsive pathways and modulation of central immune responses. Therefore, vagal afferent signaling provides an additional mechanism through which systemic inflammation can influence CNS function independently of the direct passage of inflammatory mediators across the blood–brain barrier [22,23]. The integration of these pathways results in microglial activation. Microglial cells are considered the resident macrophages of the central nervous system. Under homeostatic conditions, these cells remain quiescent and perform immune surveillance through mobile processes [20]. Due to this mobility, these cells rapidly respond to neuronal alterations and exhibit phagocytic activity. However, in response to specific inflammatory stimuli, such as an LPS-induced inflammatory condition, microglia change their phenotype, becoming activated and undergoing transcriptional changes to exert an inflammatory function [21].

**Figure 1:**
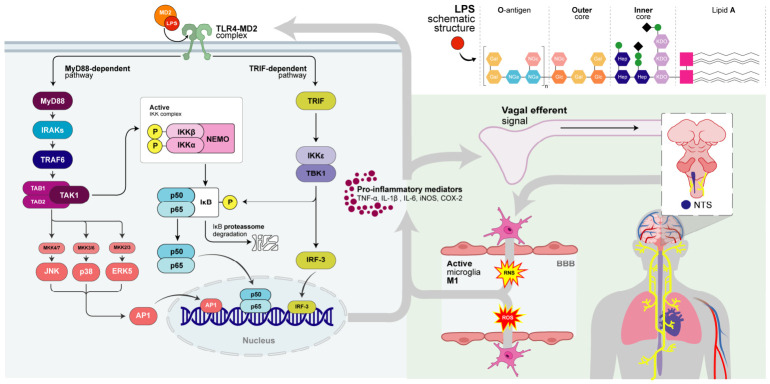
LPS recognition and TLR4-mediated signaling pathways involved in neuroinflammation associated with depression. The figure illustrates peripheral LPS recognition through TLR4 signaling and subsequent neuroimmune communication. LPS activates TLR4 pathways in peripheral immune cells and BBB-associated cells, promoting inflammatory mediator release and BBB dysfunction. These events contribute to microglial activation and amplification of neuroinflammatory responses associated with depression-like behavior. Following receptor activation, two major intracellular signaling pathways are triggered: the MyD88-dependent pathway, leading to activation of IRAKs, TRAF6, TAK1, MAPKs (JNK, p38, and ERK), and NF-κB/AP-1 transcription factors; and the TRIF-dependent pathway, involving TBK1, IKKε, and IRF3 activation. These signaling cascades promote transcription of pro-inflammatory mediators, including TNF-α, IL-1β, IL-6, iNOS, and COX-2. The figure also highlights the schematic structure of LPS and illustrates peripheral-to-central communication mediated by inflammatory signaling and vagal afferent pathways, contributing to blood–brain barrier (BBB) dysfunction, microglial activation toward the M1 phenotype, and amplification of neuroinflammatory responses implicated in the pathophysiology of depression.

This process of microglial activation begins with the recognition of these stimuli by pattern recognition receptors expressed on the membrane of these cells, especially Toll-like receptors (TLRs), with particular emphasis on TLR4, which is classically activated by LPS [22]. The binding of LPS to the TLR4–MD2 complex (the latter being a co-receptor of TLR4) triggers two major intracellular signaling pathways: the MyD88-dependent pathway, which leads to the activation of kinases such as IRAKs and TRAF6, culminating in the activation of nuclear factor kappa B (NF-κB) and MAPKs (p38, JNK, ERK), promoting the transcription of pro-inflammatory genes [24]. In contrast, the TRIF-dependent pathway induces the activation of IRF3 (interferon regulatory factor 3) and the production of interferons, contributing to the amplification of the inflammatory response [25] (Figure 1).

In addition to the production of pro-inflammatory products, activated microglia generate reactive oxygen species (ROS) and reactive nitrogen species (RNS), resulting in oxidative stress, which leads to cellular damage [26]. The combination of a pro-inflammatory and pro-oxidative environment contributes to impaired synaptic activity and decreased brain-derived neurotrophic factor (BDNF), which is closely correlated with the pathophysiology of depression, especially regarding the loss of neural plasticity and dysfunction of circuits involved in mood regulation [27,28,29]. Under conditions of chronic or persistent inflammation, such as LPS-induced models, the maintenance of a pro-inflammatory microglial state may lead to the perpetuation of neuroinflammation and the development of depressive-like behavior [30].

Based on this, neuroinflammation exerts a profound effect on central neurotransmission systems. One of the well-characterized mechanisms supporting this hypothesis involves the activation of indoleamine 2,3-dioxygenase (IDO), which is induced by pro-inflammatory cytokines such as IFN-γ, TNF-α, and IL-1β [31,32]. IDO catalyzes the degradation of tryptophan, which is the precursor of serotonin (5-HT) synthesis, redirecting its metabolism toward the kynurenine pathway [33,34]. As a consequence, a decrease in the availability of 5-HT and an increase in the production of neuroactive metabolites are observed, such as kynurenine (KYN), quinolinic acid (QUIN), which is an NMDAR agonist capable of generating glutamatergic excitotoxicity, and kynurenic acid (KYNA), an NMDAR antagonist [34,35]—which may counteract excessive glutamatergic signaling; however, abnormal accumulation or imbalance of kynurenine pathway metabolites during inflammation can disrupt physiological neurotransmission and contribute to neuronal dysfunction. This shift in metabolic routing not only compromises serotonergic neurotransmission but also impacts the glutamatergic system, establishing a critical state in which inflammation, excitotoxicity, and neural dysfunction are key pillars.

**Figure 2:**
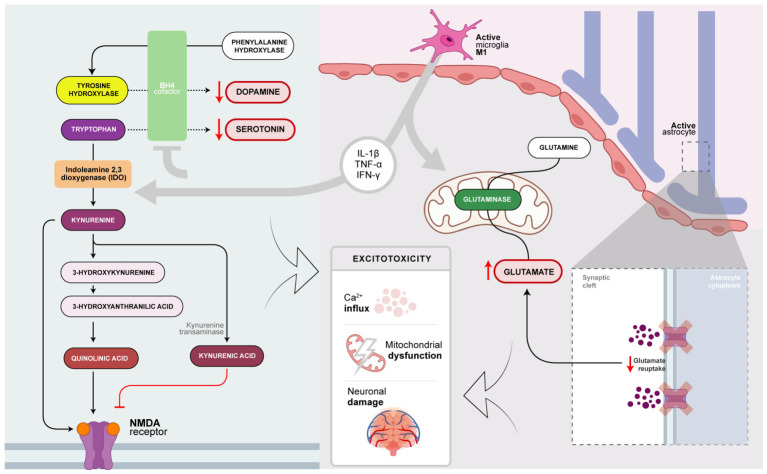
Neuroinflammatory mechanisms linking monoaminergic imbalance, kynurenine pathway activation, and glutamatergic excitotoxicity during LPS-induced depression. The figure illustrates molecular pathways through which neuroinflammation contributes to neurotransmitter dysregulation associated with depression. Pro-inflammatory cytokines released by activated M1 microglia, including IL-1β, TNF-α, and IFN-γ, stimulate activation of indoleamine 2,3-dioxygenase (IDO), promoting tryptophan metabolism toward the kynurenine pathway and increasing production of neurotoxic metabolites such as quinolinic acid. Quinolinic acid activates NMDA receptors, contributing to excessive Ca^2+^ influx, mitochondrial dysfunction, neuronal damage, and excitotoxicity. Simultaneously, inflammatory signaling reduces monoamine synthesis by decreasing tetrahydrobiopterin (BH4) availability, impairing dopamine and serotonin production. Activated microglia and astrocytic dysfunction also contribute to glutamatergic imbalance through increased glutaminase activity and reduced glutamate uptake, resulting in extracellular glutamate accumulation and amplification of excitotoxic mechanisms. Together, these pathways establish a neuroinflammatory environment associated with synaptic dysfunction and depression-like behaviors. Black arrows indicate the progression of the signaling pathway. Red lines without arrowheads indicate inhibition of a signaling pathway. Red arrows indicate increases or decreases in the levels or activity of the indicated products. Wide gray arrows are used to guide the reader through the sequential interpretation of the figure.

Glutamate is the principal excitatory neurotransmitter of the central nervous system. The inflammatory state influences this neurotransmission system, since activated microglia increase glutamate release through increased enzymatic activity of glutaminase, which is responsible for converting glutamine into glutamate [36]. Furthermore, neuroinflammation contributes to reduced glutamate reuptake by astrocytes [37]. In addition, as previously mentioned, there is excessive activation of NMDAR, especially mediated by quinolinic acid. This set of alterations favors excitotoxicity, characterized by excessive Ca^2+^ influx, mitochondrial dysfunction, and neuronal damage [38]. Beyond the imbalance of glutamatergic neurotransmission, this condition is associated with reduced synaptic plasticity and atrophy of brain regions crucial for the regulation of behavior and mood, such as the hippocampus and prefrontal cortex (Figure 2).

Indeed, inflammation also affects the dopaminergic system, especially mesolimbic circuits related to motivation and reward [39]. Pro-inflammatory mediators, such as cytokines and chemokines, may reduce dopamine synthesis through decreased availability of tetrahydrobiopterin (BH4) [40], a cofactor of tyrosine hydroxylase—the rate-limiting enzyme in dopamine synthesis, which catalyzes the conversion of L-tyrosine into L-DOPA [41]. In addition, a reduction in dopamine release in the ventral striatum is also observed [39], leading to a hypodopaminergic state in the nucleus accumbens [42], an essential area for motivation [43]. Overall, inflammation influences the monoamine system, which is directly correlated with mood regulation [44].

Taken together, these neurochemical alterations induced by systemic inflammation establish a scenario of synaptic dysfunction, in which the interplay between glutamatergic and monoaminergic pathways leads to profound neuronal consequences. In this context, LPS emerges as a fundamental experimental and mechanistic model, whereby peripheral immune activation triggers neuroinflammatory cascades that converge on these dysfunctions, thereby consolidating neuroinflammation as a central axis in the pathogenesis of depressive-like behavior.

## 4. Pharmacological Modulation of Neuroinflammatory Pathways

The pharmacological modulation of neuroinflammation has emerged as a promising strategy at the interface between pharmacology, neuroscience, and psychiatry, particularly in the context of neuropsychiatric disorders associated with immune activity. Recent evidence indicates that different classes of compounds, both synthetic and derived from natural products, are capable of interfering with central molecular pathways involved in the inflammatory response within the central nervous system (CNS), with particular emphasis on the TLR4/NF-κB/MAPK signaling pathway and the regulation of the microglial phenotype [45,46,47].

It should be noted, however, that the level of evidence supporting these compounds is heterogeneous. Most findings discussed in this section derive from preclinical studies, including in vitro assays using microglial or neuronal cell systems and in vivo animal models of neuroinflammation or depressive-like behavior. For several natural compounds, evidence remains limited to experimental models, and their effects should therefore be interpreted as mechanistic or pharmacological signals rather than as direct evidence of clinical efficacy in patients with major depressive disorder. When clinical relevance is discussed, it is framed cautiously and should be understood as a potential translational direction that requires further validation in well-designed human studies.

In this context, microglia play a central role as the primary effector cells of innate immunity in the CNS, being highly sensitive to inflammatory stimuli and capable of assuming different functional phenotypes. Classical microglial polarization (M1) is associated with the production of pro-inflammatory cytokines, such as TNF-α, IL-1β, and IL-6, in addition to reactive oxygen and nitrogen species, contributing to synaptic dysfunction and neurotoxicity. In contrast, the alternative phenotype (M2) is associated with anti-inflammatory functions, tissue repair, and neuroprotection. Thus, several pharmacological compounds have demonstrated the ability to modulate this balance, favoring the transition from a pro-inflammatory state to a neuroprotective profile [48,49].

Although the M1/M2 classification has been widely used to describe functional aspects of microglial activation, it is increasingly recognized that this framework represents a simplified model that does not fully capture the complexity of microglial responses in vivo. Recent advances in single-cell transcriptomics, spatial profiling, and multi-omics approaches have demonstrated that microglial activation occurs along a dynamic continuum of transcriptional, metabolic, and functional states rather than through a strict dichotomy between pro-inflammatory and anti-inflammatory phenotypes. Microglial responses are shaped by multiple factors, including disease context, brain region, age, cellular interactions, and the nature and duration of the inflammatory stimulus. In this con-text, activated microglia may exhibit overlapping molecular signatures, simultaneously expressing markers traditionally associated with both M1-like and M2-like states, as well as specialized phenotypes such as disease-associated microglia (DAM) and interferon-responsive microglia [50]. Therefore, in the context of LPS-induced neuroinflammation, the M1/M2 terminology should be interpreted as a conceptual framework for describing functional tendencies rather than as discrete and irreversible cellular identities. This perspective highlights microglial plasticity as a key determinant of inflammatory progression, resolution, and therapeutic response [51].

Activation of Toll-like receptor 4 (TLR4), particularly in CNS-resident immune cells such as microglia, represents one of the primary triggers of the neuroinflammatory response. Following the recognition of molecular patterns associated with pathogens or cellular damage, TLR4 activates MyD88-dependent intracellular cascades, resulting in the activation of the transcription factor NF-κB and kinase pathways such as MAPK (p38, ERK, and JNK). These pathways regulate the expression of inflammatory genes and are directly implicated in the pathophysiology of inflammation-associated depression [52]. Several preclinical studies have demonstrated that bioactive compounds can inhibit this signaling in experimental models. Gypenosides, for example, were able to reduce depressive-like behaviors in animal models by suppressing the TLR4/MyD88/NF-κB pathway and promoting the negative modulation of the microglial state [45]. Similarly, ginsenoside Rh2 demonstrated antidepressant-like effects in offspring of mice exposed to maternal infection, reducing microglial activation through inhibition of the HMGB1/TLR4/NF-κB axis [46].

Furthermore, the MAPK pathway also plays a relevant role in amplifying the inflammatory response, being associated with the production of pro-inflammatory mediators and the regulation of cell survival. Compounds such as polyunsaturated fatty acids, including ω-3 DPA, have demonstrated the ability to attenuate neuroinflammation by inhibiting NF-κB and MAPK p38 activation, in addition to promoting microglial polarization toward the M2 phenotype and activating neurotrophic pathways such as BDNF/PI3K/AKT [47]. These findings reinforce the importance of simultaneously modulating multiple signaling pathways as a therapeutic strategy.

Regarding natural compounds, there is growing evidence that phytoconstituents exert pleiotropic effects on neuroinflammation. Flavonoids such as kaempferol and phenolic compounds such as punicalagin have demonstrated the ability to reduce the production of inflammatory cytokines and the expression of iNOS and COX-2, mainly through inhibition of the NF-κB and MAPK pathways [53,54]. Likewise, ferulic acid derivatives have shown anti-inflammatory effects in human microglial cells by directly modulating NF-κB activity [49]. These compounds also exhibit antioxidant properties, contributing to the reduction in oxidative stress, which is closely related to neuroinflammation [55].

**Figure 3:**
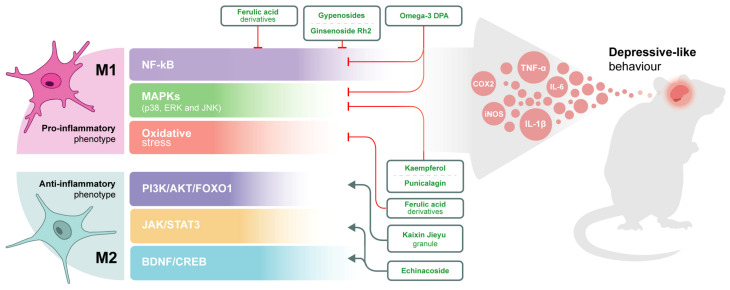
Microglial activation states and pharmacological modulation of neuroinflammatory signaling pathways in LPS-induced depression-like behavior. The figure illustrates the functional spectrum of microglial responses during neuroinflammation. Although traditionally categorized into M1-like and M2-like phenotypes, current evidence indicates that microglial activation represents a dynamic continuum of context-dependent states. Pro-inflammatory signaling pathways, including NF-κB and MAPKs, contribute to inflammatory mediator production, whereas regulatory and reparative pathways involving PI3K/AKT/FOXO1, JAK/STAT3, and BDNF/CREB signaling are associated with resolution and neuroprotective responses. Bioactive compounds may modulate this functional balance by shifting microglial activity toward less inflammatory and more homeostatic states. The figure also highlights bioactive compounds, including ferulic acid derivatives, gypenosides, ginsenoside Rh2, omega-3 DPA, kaempferol, punicalagin, Kaixin-Jieyu granules, and echinacoside, which modulate these molecular pathways by suppressing pro-inflammatory signaling and promoting anti-inflammatory responses, thereby attenuating neuroinflammation and depression-like behaviors.

Another relevant aspect is the modulation of additional pathways involved in the neuroimmune response, such as PI3K/AKT/FOXO1 and JAK/STAT. Traditional formulations, such as Kaixin Jieyu Granule, demonstrated the ability to reduce depressive behaviors in animal models through positive regulation of the TLR4/PI3K/AKT/FOXO1 pathway, highlighting the complexity of the molecular interactions involved [56]. Likewise, compounds such as echinacoside exhibited antidepressant effects associated with the regulation of microglial polarization and modulation of the JAK1/STAT3 pathway, in addition to promoting hippocampal neurogenesis via the BDNF/CREB axis [57] (Figure 3).

Within the scope of synthetic drugs, classical antidepressants have also been implicated in the modulation of neuroinflammation. Experimental evidence suggests that selective serotonin reuptake inhibitors (SSRIs) can reduce microglial activation and modulate cytokine networks, suggesting mechanisms that may complement their established monoaminergic effects [58,59,60]. Finally, it should be emphasized that the formation of bioactive metabolites and interactions with multiple molecular targets are important determinants of the efficacy of these compounds. The modulation of neuroinflammation frequently involves integrated mechanisms, including the regulation of cytokines, inhibition of pro-inflammatory pathways, promotion of neuroprotective pathways, and reduction in oxidative stress.

Overall, these findings indicate that pharmacological modulation of neuroinflammatory pathways may represent a promising mechanistic strategy for attenuating inflammation-associated depressive-like phenotypes in experimental settings [1,3]. Nevertheless, the therapeutic relevance of many of these compounds remains preliminary, particularly for natural products whose evidence is largely restricted to in vitro assays or animal models. Future studies should better define dose–response relationships, pharmacokinetic properties, blood–brain barrier permeability, safety profiles, and clinical efficacy before these compounds can be considered translational candidates for depression treatment [2,3]. Therefore, the compounds discussed in this section should be interpreted primarily as tools for understanding neuroimmune regulation and as potential leads for further investigation, rather than as established therapeutic interventions [61]. For clarity and comparative purposes, Table 1 provides a concise overview of the principal signaling pathways discussed in this review, together with their major molecular targets, representative therapeutic compounds, and the corresponding pharmacological effects associated with experimental modulation of LPS-induced neuroinflammation and depression-like behavior.

## 5. Involvement of the NLRP3 Inflammasome in the Amplification of the LPS-Mediated Inflammatory Response in Depression

Although the initial activation triggered by LPS through the TLR4/NF-κB axis in innate immune cells, particularly microglia within the CNS, represents a central event in endotoxin-induced neuroinflammation, growing evidence indicates that the maintenance and amplification of this inflammatory response depend on the subsequent activation of intracellular pathways capable of perpetuating the inflammatory state. In this context, the NLRP3 inflammasome emerges as one of the most relevant mechanisms. Inflammasome activation constitutes a stage of the immune response that amplifies the signal induced by LPS, promoting a transition from acute inflammatory signaling to a persistent neuroinflammatory state associated with the development of behavioral alterations consistent with depression [11,58,61].

The NLRP3 inflammasome belongs to the family of cytoplasmic pattern recognition receptors, particularly the NOD-like receptor (NLR) family, functioning as an intracellular sensor of signals associated with damage and homeostatic disturbances [70,71]. In contrast to classical pattern recognition receptors capable of recognizing specific pathogen structures, NLRP3 exhibits the ability to respond to cellular signals, including ROS, mitochondrial dysfunction, ionic flux alterations, among other disturbances [82]. This capability makes the inflammasome particularly important in inflammatory conditions, including psychiatric disorders associated with chronic inflammation, such as depression.

Its activation occurs in two stages: a priming phase and an activation phase itself. The first step is frequently induced by LPS binding to the TLR4/MD2 complex, primarily activating the MyD88-dependent pathway and culminating in NF-κB activation. This transcription factor promotes increased gene expression of essential components for inflammasome formation, including pro-IL-1β, pro-IL-18, and NLRP3 [83]. Thus, LPS initially acts by preparing the cell for an inflammatory response that will subsequently become exacerbated. However, increased expression of inflammasome components alone is not sufficient for its functional assembly [83].

A second signal is required to promote NLRP3 oligomerization and formation of the active multiprotein complex. Several intracellular mechanisms associated with LPS can provide this second stimulus. Among the most extensively studied mechanisms, excessive ROS generation stands out [70,71].The increase in mitochondrial ROS promotes oxidation of intracellular proteins and alterations in mitochondrial integrity, favoring the release of oxidized mitochondrial DNA and cardiolipin, molecules recognized as important inducers of NLRP3 activation [84,85,86]. In parallel, ROS induce dissociation of TXNIP (thioredoxin-interacting protein) from its complex with thioredoxin, an important antioxidant system responsible for maintaining redox homeostasis [87]. Under physiological conditions, TXNIP remains bound to reduced thioredoxin, maintaining a functionally inactive state. However, during states of intense oxidative stress induced by LPS, excessive ROS promote TRX oxidation, reducing its affinity for TXNIP and favoring its dissociation. Once released, TXNIP functions as an intracellular sensor of oxidative stress by translocating and binding directly to the leucine-rich repeat (LRR) domain of NLRP3 [88]. This interaction favors conformational changes necessary for inflammasome oligomerization and subsequent recruitment of the ASC–procaspase-1 complex. Thus, TXNIP constitutes an important molecular link between redox dysfunction and inflammatory activation, converting oxidative stress signals into amplified pro-inflammatory responses [89].

Beyond oxidative stress, alterations in intracellular ionic balance represent another critical mechanism for NLRP3 activation, with potassium efflux being the best-characterized mechanism converging on inflammasome activation [82]. During inflammatory processes, extracellular ATP released by injured cells or activated glial cells may stimulate P2X7 purinergic receptors expressed in microglia [90]. P2X7 is a trimeric ionotropic receptor whose activation requires high ATP concentrations. ATP binding promotes conformational changes in the receptor culminating in the opening of a non-selective cationic channel, allowing Na^+^ and Ca^2+^ influx, this last one being responsible for a number of P2X7 receptor-induced responses, as the activation of some kinases, like protein kinase C (PKC) and calcium-calmodulin kinase II (CaMKII), which phosphorylates and activates phosphoinositide 3-kinase (PI3K), extracellular signal-regulated kinases 1/2 (ERK1/2), protein kinase B (AKT) and glycogen synthase kinase 3 (GSK3) [91], and, most importantly, K^+^ efflux [92].

Physiologically, elevated cytosolic K^+^ concentrations appear to exert an inhibitory effect on NLRP3 complex assembly. Thus, K^+^ efflux removes this intracellular repressive state, allowing recruitment of regulatory proteins involved in inflammasome activation. Recent studies demonstrate that this reduction in intracellular K+ concentration favors recruitment of NEK7 (NIMA-related kinase 7), considered an essential component for NLRP3 activation. NEK7 acts as a structural regulator of the inflammasome [70,82,93]. Under low potassium concentration environments, NEK7 directly interacts with both the LRR domain and the NACHT region of NLRP3, stabilizing conformational alterations necessary for its oligomerization. This interaction favors self-association of multiple NLRP3 molecules and subsequent recruitment of the adaptor protein ASC (apoptosis-associated speck-like protein containing CARD) [88,94]. ASC acts as a central molecular platform for inflammasome assembly, functioning as a structural bridge between the sensor receptor and effector proteins. Structurally, ASC contains two distinct domains: an N-terminal PYD domain and a C-terminal CARD (caspase recruitment domain) domain [82]. Following recruitment to NLRP3, multiple ASC molecules undergo polymerization and form highly organized filamentous structures culminating in the formation of large cytoplasmic aggregates known as ASC specks [95]. These complexes function as centers capable of concentrating inflammatory components and amplifying response efficiency [96].

**Figure 4:**
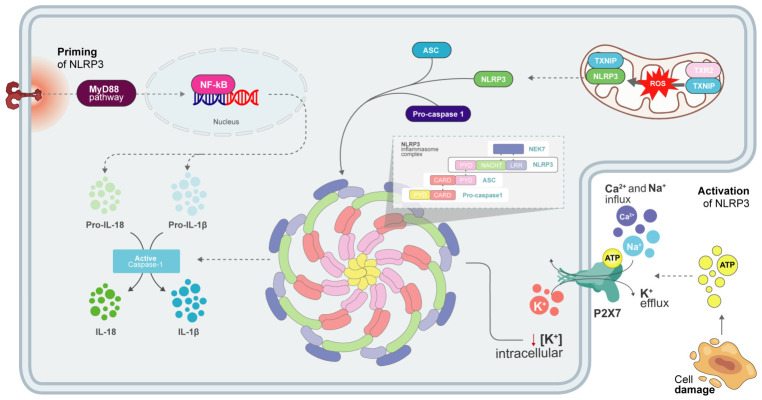
Mechanisms involved in NLRP3 inflammasome activation and amplification of the LPS-induced inflammatory response. LPS-mediated activation of the TLR4/MyD88 pathway promotes NF-κB activation, initiating the priming phase of the NLRP3 inflammasome through increased transcription of inflammasome-related components, including NLRP3, pro-IL-1β, and pro-IL-18. Subsequently, intracellular stress signals provide the second activation signal required for inflamassome assembly. Mitochondrial dysfunction induced by oxidative stress promotes excessive production of reactive oxygen species (ROS), favoring TXNIP dissociation from thioredoxin (TRX) and subsequent interaction with NLRP3. In parallel, extracellular ATP released following cellular damage activates P2X7 receptors, inducing K^+^ efflux and reducing intracellular potassium concentration, thereby promoting NEK7 recruitment and NLRP3 oligomerization. Assembly of the inflammasome complex facilitates recruitment of ASC and procaspase-1, leading to caspase-1 activation and maturation of IL-1β and IL-18. Collectively, these processes amplify inflammatory signaling and contribute to the maintenance of persistent neuroinflammation associated with depressive pathophysiology. Arrows pointing downwards indicate a decrease in intracellular potassium concentration. The other arrows were used to guide the reader.

Formation of ASC specks allows recruitment of multiple procaspase-1 molecules through homotypic CARD–CARD interactions [97]. Under physiological conditions, procaspase-1 remains dispersed throughout the cytoplasm in a catalytically inactive state. However, its elevated local concentration within the inflammasome complex promotes a mechanism termed “proximity-induced autocatalysis” [98]. In this process, the spatial proximity of multiple procaspase-1 molecules favors their proteolytic self-cleavage, generating catalytic subunits that reorganize to form mature functional caspase-1 [99]. Active caspase-1 promotes proteolytic cleavage of pro-IL-1β and pro-IL-18, formed during the aforementioned priming phase, into their biologically active forms, IL-1β and IL-18 (Figure 4).

Furthermore, caspase-1 also promotes cleavage of the protein gasdermin D (GSDMD), currently recognized as the main molecular executor of pyroptosis. Structurally, GSDMD contains two domains: an N-terminal domain with intrinsic cytotoxic activity and an autoinhibitory C-terminal domain that, under physiological conditions, maintains the protein in a functionally inactive state. Caspase-1-mediated cleavage disrupts this intramolecular interaction and releases the active N-terminal domain [100]. Following its release, multiple GSDMD-N fragments rapidly translocate to the plasma membrane, where they interact with anionic phospholipids, particularly phosphatidylinositol and cardiolipin [101]. These molecules oligomerize and form large transmembrane pores approximately 15 nm in diameter, profoundly disrupting cellular homeostasis by allowing uncontrolled ion flux, including Ca^2+^ influx, water entry, progressive cellular swelling, and loss of osmotic integrity [102,103]. These pores permit extracellular release of IL-1β and IL-18 and trigger a specific inflammatory process termed pyroptosis [104,105]. Pyroptosis represents a highly inflammatory form of programmed cell death characterized by membrane rupture, cellular swelling, and massive release of pro-inflammatory intracellular contents, including ATP, HMGB1, IL-1α, and additional DAMPs [106]. Unlike classical apoptosis, pyroptosis intensely amplifies local inflammatory signals, generating a feed-forward circuit that perpetuates immune activation [104].

Additionally, during LPS-induced neuroinflammation, sustained microglial activation and increased cellular metabolic demand favor alterations in mitochondrial dynamics, including excessive fragmentation, loss of mitochondrial membrane potential (ΔΨm), alterations in fusion and fission processes, and impairment of the electron transport chain [107,108]. These alterations promote electron leakage primarily at complexes I and III of the respiratory chain, increasing the generation of mitochondrial reactive oxygen species (mitochondrial ROS, mtROS). Among the mechanisms involved in NLRP3 activation, increased mtROS constitutes one of the intracellular signals most consistently associated with its activation [72].

In parallel, cardiolipin may undergo redistribution to outer regions of the organelle following cellular damage, functioning as an anchoring platform for the inflammasome. Studies further suggest the involvement of the adaptor protein MAVS (mitochondrial antiviral signaling protein), localized in the outer mitochondrial membrane, in spatially approximating damaged mitochondria and inflammasome components, thereby facilitating its activation [109,110]. However, the relationship between mitochondria and the inflammasome is not unidirectional. Following activation, the NLRP3 complex itself progressively aggravates mitochondrial injury [111]. Active caspase-1, in addition to processing pro-inflammatory cytokines, may indirectly interfere with mitochondrial homeostasis through increased inflammatory mediators and intracellular oxidative damage. IL-1β and IL-18 amplify glial activation and increase local production of TNF-α, nitric oxide, and ROS, intensifying cellular energetic impairment [112,113].

Within the central nervous system, such alterations assume particular relevance due to the high energetic demand of neurons and glial cells. Maintenance of neurotransmission and synaptic plasticity strongly depends on mitochondrial integrity [114]. Therefore, sustained mitochondrial damage may directly affect neurobiological processes involved in the pathophysiology of depression, including neuroplasticity, hippocampal neurogenesis, and neurotransmitter signaling [112,115]. Consequently, a feed-forward circuit is established in which mitochondrial damage increases NLRP3 activation, while inflammasome activation progressively aggravates mitochondrial dysfunction itself [116]. This cycle creates a state of mutual dependence between chronic inflammation and cellular bioenergetic impairment, contributing to the maintenance of persistent neuroinflammation observed in LPS-induced depression models.

In experimental models of LPS-induced depression, a significant increase in NLRP3 [116] and ASC expression has been identified in brain regions associated with affective behavior, particularly the hippocampus and prefrontal cortex [70,117]. Moreover, NLRP3 knockout models exhibited attenuation of depression-like behavior induced by LPS-mediated neuroinflammation [118]. These findings suggest that the NLRP3 inflammasome acts not only as a secondary mediator of the response to LPS but also as a central amplifier of neuroinflammation capable of transforming primary immune activation into a sustained inflammatory state associated with depressive pathophysiology. Thus, pharmacological modulation of this pathway emerges as a promising therapeutic strategy for depressive disorders associated with systemic and neuroimmunological inflammation.

Pharmacological strategies have been investigated to inhibit NLRP3 activation [119]. Among the most studied compounds, MCC950 represents a selective NLRP3 inhibitor capable of suppressing inflammasome assembly and reducing caspase-1 activation and IL-1β maturation [120]. Experimental studies demonstrate that MCC950 attenuates neuroinflammatory responses and improves depression-like behaviors in animal models exposed to inflammatory stimuli, supporting the relevance of NLRP3 as a potential therapeutic target [121,122]. However, despite its high experimental specificity, clinical translation of MCC950 has been limited by safety concerns observed in early investigations, highlighting the need for improved NLRP3 inhibitors with favorable pharmacokinetic profiles [120].

Other approaches include indirect modulation of NLRP3 activation through regulation of upstream pathways. Pharmacological inhibition of TLR4/NF-κB signaling, oxidative stress pathways, and mitochondrial dysfunction has been shown to reduce NLRP3 inflammasome activation in experimental models. In addition, natural bioactive compounds with antioxidant and anti-inflammatory properties may attenuate NLRP3-dependent inflammatory responses by decreasing reactive oxygen species production, preserving mitochondrial homeostasis, and restoring redox balance [123,124].

## 6. LPS-Induced Oxidative Stress and Its Role in Depressive Behavior

### 6.1. LPS and NADPH Oxidase

Lipopolysaccharide (LPS), an endotoxin component present in the outer membrane of Gram-negative bacteria, is widely used in translational models to mimic neuroinflammation and depression-like behaviors in rodents [11,15]. Recognition of LPS occurs predominantly through Toll-like receptor 4 (TLR4) pattern recognition receptors, which are expressed in peripheral and central innate immune cells, including macrophages, monocytes, dendritic cells, and Schwann cells [88,125].

Within the central nervous system (CNS), TLR4 is expressed in several cell types involved in neuroimmune communication, including endothelial cells associated with the blood–brain barrier (BBB) and resident immune cells such as microglia. Although microglia are not structural components of the BBB and do not directly contact circulating blood under physiological conditions, they can be activated by peripheral inflammatory signals transmitted through BBB-associated cells, circulating cytokines, and inflammation-induced alterations in barrier integrity. TLR4 consists of a leucine-rich repeat (LRR)-containing extracellular domain connected to an intracellular Toll/IL-1 receptor (TIR) domain responsible for signal transduction [126]. Although circulating LPS exhibits limited capacity to cross the BBB, its interaction with endothelial and perivascular microglial receptors is sufficient to trigger an intracellular signaling cascade mediated by nuclear factor kappa B (NF-κB), ultimately leading to microglial activation toward the pro-inflammatory M1 phenotype and the subsequent release of pro-inflammatory cytokines, including tumor necrosis factor-alpha (TNF-α), IL-6, and interleukin-1 beta (IL-1β) [127].

Recognition and activation of TLR4 by LPS occur through a sequence of coordinated steps until formation of the final signaling complex. Initially, LPS-binding protein (LBP) binds to the LPS monomer and transfers it to cluster of differentiation 14 (CD14) through electrostatic interactions. CD14, in turn, is membrane-anchored via a glycosylphosphatidylinositol (GPI) anchor. Through the adaptor protein myeloid differentiation protein-2 (MD-2), the LPS/CD14 complex associates with TLR4, forming the LPS/MD-2/TLR4 complex, inducing TIR-domain dimerization and receptor activation [88,126,128].

Once TIR-domain dimerization is established, recruitment of the myeloid differentiation factor 88-dependent adaptor protein (MyD88) occurs. MyD88 recruitment triggers sequential phosphorylation of IL-1 receptor-associated kinases (IRAK4 and IRAK1/2) and subsequent activation of tumor necrosis factor receptor-associated factor 6 (TRAF6), ultimately promoting both nuclear translocation of NF-κB for transcription of pro-inflammatory genes such as IL-1β, TNF-α, IL-6, NLRP3, iNOS, and COX-2, as well as parallel cytoplasmic enzymatic signaling pathways [128,129].

In addition to NF-κB activation, another downstream mechanism is particularly relevant to activation of the LPS/TLR4 axis within the brain parenchyma. This mechanism involves cytoplasmic oxidative responses mediated by NADPH oxidase (NOX), particularly the NOX2 isoform, which is highly expressed in reactive microglial cells. Under basal conditions, NOX2 remains inactive, with its heterodimeric catalytic domain anchored to the plasma membrane and its regulatory subunits dispersed within the cytosol. However, under inflammatory conditions and persistent stress, such as LPS-induced TLR4/TRAF6 activation, stimulation promotes translocation of cytosolic NOX subunits to the cellular membrane, significantly increasing expression, assembly, and activity of the NOX2 enzymatic complex [63,64].

The classical pathophysiological role of NOX resides in cleaving cellular reduced nicotinamide adenine dinucleotide phosphate (NADPH) to transfer electrons to molecular oxygen, converting NADPH into superoxide anion radicals (O_2_•^−^). Sustained production of free radicals by NOX acts as an initiating trigger for establishing a neurotoxic environment and is directly associated with persistent oxidative stress and induction of depression-like behaviors [64,65].

Paradoxically, the molecular dynamics of NADPH play a dual and crucial role in redox homeostasis and glial morphology. While the NOX enzymatic complex consumes intracellular NADPH to generate inflammatory reactive oxygen species (ROS), free NADPH serves as one of the major cofactors utilized by endogenous antioxidant systems, playing an essential role in regeneration of reduced glutathione (GSH) through glutathione reductase activity. Given this duality, recent studies have demonstrated that exogenous NADPH treatment attenuates LPS-induced oxidative stress and mitigates depression-like behaviors in rodents [66].

This neuroprotective effect is associated with reversal of morphological alterations in reactive microglia, reducing expression intensity of the activation marker Iba1, emphasizing that modulation of NADPH availability and NOX activity constitutes a promising therapeutic strategy for managing inflammation-induced depression [66].

### 6.2. LPS-Induced ROS and RNS Production

Reactive oxygen species (ROS) and reactive nitrogen species (RNS), although functioning under tightly regulated concentrations as physiological second messengers, contribute to chronic oxidative and nitrosative stress following disruption of cerebral redox homeostasis. Since they are induced and sustained by the response to lipopolysaccharide (LPS), these species act as important mediators of cellular damage in the pathophysiology of affective disorders, particularly major depressive disorder (MDD) [130].

Within the context of oxidative stress, three brain regions stand out for the neurochemical study of depression and anxiety: the prefrontal cortex (PFC), hippocampus, and striatum. The PFC represents a crucial neural region involved in mood regulation, memory, and cognitive functions, being directly implicated in the neurobiology of depression [131]. The hippocampus, in turn, significantly contributes to the development of this disorder because it constitutes an essential region for learning and memory, functions that become impaired during depressive states [132]. Additionally, the striatum plays a central role in numerous behaviors, ranging from motor control to action selection and reward-based learning [133].

These three anatomical regions represent major targets of oxidative stress and undergo intense lipid peroxidation during sustained inflammatory processes, such as those observed in depression. This occurs, in part, due to their high concentrations of iron and polyunsaturated fatty acids within synaptic membranes, factors that contribute to excessive free radical generation and lipid peroxidation [134,135].

With LPS-induced inflammatory stimulation promoting the production of pro-inflammatory cytokines, nitric oxide (NO) levels increase in both cerebral and peripheral tissues due to direct upregulation of inducible nitric oxide synthase (iNOS). Consequently, nitrosative stress (NS) is synergistically enhanced through the activity of neuronal nitric oxide synthase (nNOS), which is widely distributed throughout the CNS. Therefore, this substantial increase in NO and NS results in damage to glial cells and neurons within the PFC, hippocampus, and striatum. In parallel, endogenous defense mechanisms become impaired, including the action of the tripeptide glutathione (GSH), which exerts antioxidant activity through its sulfhydryl group to mitigate oxidative stress, along with reduced activity of enzymes such as superoxide dismutase (SOD) and catalase [117,135,136].

As a direct consequence of oxidative damage and reduction in antioxidant defenses, massive release of malondialdehyde (MDA) and carbonylated proteins occurs as a result of lipid peroxidation [64,65,135]. Clinically, MDA has been proposed as a novel sensitive predictive biomarker for depression, given its elevated plasma levels in patients with major depressive disorder [137].

Beyond direct structural damage, LPS-induced oxidative stress acts as a potent negative modulator of intracellular signaling cascades crucial for synaptic plasticity. Excessive free radical accumulation blocks activation of ERK1/2 kinases as well as the PI3K/Akt/mTOR cell survival pathway [11]. Suppression of these pathways prevents activation of the transcription factor CREB and dysregulates nuclear factor erythroid 2-related factor 2 (Nrf2), ultimately reducing expression of brain-derived neurotrophic factor (BDNF) and its high-affinity receptor, TrkB. Consequently, neuronal plasticity becomes compromised, resulting in reduced synaptogenesis and dendritic atrophy, alterations closely associated with hippocampal and cortical dysfunction observed in depression and favoring the development of depression-like behaviors [138].

In parallel, excessive accumulation of ROS and RNS also acts as an important intracellular pro-inflammatory signaling mechanism, promoting activation of the NLRP3 inflammasome and sustaining the neuroinflammatory response. Within the intracellular environment, elevated ROS levels induce production of oxidized mitochondrial DNA (oxidized mtDNA), which promotes dissociation of thioredoxin-interacting protein (TXNIP) from its endogenous ligand [139]. Together, oxidized mtDNA and free TXNIP function as signaling molecules for assembly and activation of the NLRP3 inflammasome in the hippocampus [117,140].

Once activated, the NLRP3 inflammasome cleaves caspase-1, culminating in processing and sustained release of highly inflammatory peripheral and central cytokines, including interleukin-1 beta (IL-1β) and interleukin-6 (IL-6), which contribute to the systemic inflammatory profile observed in patients with major depression [64,141].

Ultimately, this process promotes recruitment of inflammatory enzymes such as cyclooxygenase-2 (COX-2), which stimulates prostaglandin E2 (PGE_2_) production, increasing generation of pro-inflammatory mediators and prolonging as well as sustaining microglial activation [135]. These inflammatory markers disrupt tissue integrity and cross the blood–brain barrier, thereby consolidating chronic neuroinflammation and becoming key potential therapeutic targets for future strategies in the management of affective disorders.

### 6.3. LPS-Induced Mitochondrial Dysfunction in Microglia and Neuronal Cells

The maintenance of mitochondrial function is essential for neuronal homeostasis due to the high energetic demand of the central nervous system [111]. Mitochondria produce ATP through oxidative phosphorylation, a process in which electrons derived from NADH and FADH2 pass through complexes I–IV of the electron transport chain, promoting proton pumping across the inner mitochondrial membrane and generating the electrochemical gradient utilized by ATP synthase for ATP synthesis [73,74,142]. During this process, small amounts of reactive oxygen species (ROS) are physiologically produced and participate in cellular signaling mechanisms. However, when produced in excess, these molecules promote oxidative damage to proteins, lipids, and cellular components and may trigger autophagy, apoptosis, necrosis, and inflammation [142,143,144].

In this context, exposure to LPS induces intense microglial activation and increased ROS production, promoting significant mitochondrial dysfunction [75]. Excessive ROS impairs proteins of the mitochondrial respiratory chain, especially complexes I and IV, reducing electron flow and decreasing the efficiency of oxidative phosphorylation [76]. In addition, superoxide-derived radicals promote peroxidation of cardiolipin (CL) and phosphatidylethanolamine (PE), phospholipids essential for the stability of the inner mitochondrial membrane. This process compromises the structural and functional integrity of respirasomes and ATP synthase, aggravating respiratory chain dysfunction [77].

Consequently, impairment of proton pumping by respiratory complexes occurs, leading to dissipation of the electrochemical gradient and loss of mitochondrial membrane potential (ΔΨm), directly reducing oxidative phosphorylation and ATP production. Reduced ATP synthesis compromises highly energy-dependent processes in the central nervous system, including maintenance of neuronal membrane potential, neurotransmission, and synaptic plasticity, thereby favoring progressive neuronal dysfunction. Studies demonstrate that these bioenergetic alterations are associated with the development of depression-like behaviors in LPS-induced experimental models, including anhedonia, increased immobility time, and hippocampus-related cognitive alterations [61,78,145,146].

In addition to bioenergetic alterations, LPS also impairs mitochondrial dynamics through activation of the TLR4/NF-κB pathway, increasing DRP1 phosphorylation and reducing mitochondrial fusion proteins, such as Mfn1 and Mfn2, favoring a pro-fission state and intense mitochondrial fragmentation. Fragmented mitochondria exhibit lower respiratory efficiency, greater electron leakage, and increased production of H_2_O_2_ and nitrite, perpetuating cellular oxidative stress. This mitochondrial fragmentation is associated with the metabolic reprogramming of reactive microglia and the maintenance of a pro-inflammatory phenotype capable of amplifying neuroinflammation related to LPS-induced depression [105].

In parallel, LPS reduces mitochondrial biogenesis by decreasing the expression of PGC-1α, NRF1, and Tfam, key factors involved in mtDNA replication and mitochondrial protein synthesis, compromising the renewal and functional maintenance of these organelles. Reduced mitochondrial biogenesis aggravates cellular energy deficits and contributes to the persistence of mitochondrial dysfunction observed in inflammation-induced depression models. Importantly, pharmacological modulation of this pathway has been shown to reduce depression-like behavioral alterations in models exposed to LPS and chronic mild stress, reinforcing the relationship between mitochondrial bioenergetics and depressive pathophysiology [79].

Experimental models using LPS demonstrate that systemic inflammatory activation induces profound mitochondrial biochemical alterations in the brain [11,146]. Persistent microglial activation promotes a significant increase in the production of reactive oxygen and nitrogen species, favoring intense mitochondrial oxidative stress [80]. In addition, mitochondrial injury leads to the release of intracellular pro-inflammatory signals, such as oxidized mtDNA, cardiolipin, and mitochondrial ROS, which amplify the neuroimmune response induced by LPS [81]. These mechanisms promote sustained neuroinflammatory activation in brain regions involved in emotional regulation, such as the hippocampus and prefrontal cortex, contributing to behavioral alterations associated with depression.

Another relevant mechanism involves activation of the NLRP3 inflammasome. Excessive mitochondrial ROS production acts as an important signaling mechanism for activation of this multiprotein complex, promoting IL-1β and IL-18 maturation and induction of pyroptosis. This process intensifies neuroinflammation and contributes to functional impairment of brain regions associated with emotional regulation, particularly the hippocampus. Corroborating these findings, studies demonstrate that improvement of mitochondrial function reduces NLRP3 inflammasome activation and attenuates LPS-induced depressive behaviors, highlighting the direct participation of mitochondrial dysfunction in the pathophysiology of inflammation-associated depression [116].

Activation of the TLR4 pathway in the brain appears to represent one of the major mechanisms responsible for linking peripheral inflammation to depression-like behavioral alterations. TLR4 stimulation by LPS promotes sustained microglial activation, increased blood–brain barrier permeability, and intensification of cerebral oxidative stress. These alterations favor progressive mitochondrial dysfunction and impairment of monoaminergic neurotransmission, especially serotonergic and dopaminergic signaling, mechanisms classically associated with the pathophysiology of depression [147].

Additionally, endogenous bacterial endotoxins also appear to contribute to neuroinflammatory alterations associated with mitochondrial dysfunction. LPS derived from *Porphyromonas gingivalis* has been shown to induce increased ROS production, mitochondrial oxidative damage, and oxidative stress-dependent neuroinflammatory activation [80]. These findings reinforce the hypothesis that chronic peripheral inflammatory processes may directly impact cerebral bioenergetics and promote behavioral alterations associated with depression [136].

The translational relevance of experimental models of LPS-induced depression has been reinforced by meta-analyses demonstrating high consistency among findings related to mitochondrial dysfunction in these animal models. Among the main mechanisms observed are increased oxidative stress, impaired oxidative phosphorylation, reduced ATP production, and alterations in cellular redox homeostasis, events directly associated with the development of neuroinflammation and depression-like behavioral alterations [11].

Therefore, current evidence demonstrates that LPS promotes profound mitochondrial biochemical alterations, including increased ROS production, respiratory chain impairment, reduced ATP production, mitochondrial fragmentation, reduced mitochondrial biogenesis, and activation of the NLRP3 inflammasome [79,116]. Thus, these events establish a persistent cycle of neuroinflammation capable of compromising neuronal plasticity, altering circuits involved in emotional regulation, and promoting the development of depression-like behaviors.

### 6.4. LPS Leading to Peroxynitrite Formation in Microglia and Neuronal Cells

In addition to ROS-mediated oxidative stress, LPS-induced neuroinflammation also promotes a significant increase in the production of reactive nitrogen species (RNS), establishing a state of nitrosative stress associated with the development of depression-like behaviors [11,148]. Following recognition of LPS by TLR4, activation of NF-κB-dependent pro-inflammatory pathways occurs in microglia and astrocytes, promoting increased expression of the inducible nitric oxide synthase (iNOS) enzyme and a consequent elevation in nitric oxide (NO) production [67].

In parallel, microglial activation induces increased production of superoxide anion (O_2_•^−^), mainly through enzymes such as NADPH oxidase and the mitochondrial alterations previously described. Under inflammatory conditions, NO rapidly reacts with O_2_•^−^ to form peroxynitrite (ONOO^−^), one of the most cytotoxic reactive nitrogen species in the neuroinflammatory context. Studies in BV2 microglial cells demonstrated that iNOS plays a central role in the formation of peroxynitrite-mediated protein radicals following LPS exposure, highlighting the relevance of the nitrergic pathway in LPS-induced cellular oxidative damage [149].

Furthermore, peroxynitrite may undergo protonation and form peroxynitrous acid (ONOOH), a highly unstable molecule that generates extremely reactive secondary radicals, such as hydroxyl radical (OH•) and nitrogen dioxide (NO_2_•), amplifying intracellular oxidative and nitrosative damage [150,151]. Thus, RNS intensify previously discussed mechanisms of mitochondrial damage, aggravating bioenergetic alterations associated with LPS-induced neuroinflammation.

Besides the bioenergetic impairment, peroxynitrite directly participates in the amplification of the cerebral inflammatory response. Evidence demonstrates that reactive nitrogen species can activate the NLRP3 inflammasome, promoting caspase-1 activation, IL-1β and IL-18 maturation, and induction of pyroptosis, thereby intensifying neuroinflammation. Although the study was conducted in an experimental model of amyotrophic lateral sclerosis, the authors demonstrated an association between nitrosative stress, mitochondrial damage, and inflammatory activation, mechanisms also observed in LPS-induced neuroinflammatory models [150].

Experimental studies have demonstrated that LPS administration promotes increased iNOS expression, elevated NO production, and intensification of cerebral oxidative damage, events associated with the development of depression- and anxiety-like behaviors [146,152]. Corroborating these findings, researchers observed that pharmacological inhibition of nitric oxide synthase exerted an antidepressant effect in animals subjected to LPS administration, suggesting that reduced NO production and, consequently, decreased peroxynitrite formation attenuate neuroinflammation and cerebral oxidative damage [153].

Several studies reinforce this relationship by demonstrating that compounds capable of reducing oxido-nitrosative stress also promote behavioral improvement in LPS-induced depression models. Honokiol reduced inflammatory and nitrosative markers, attenuating depressive behavior in LPS-treated mice [68]. Similarly, the PPAR-α agonist WY-14643 reduced neuroinflammation and oxido-nitrosative stress, promoting improvement in depression-like behaviors [69].

Corroborating these findings, meta-analyses involving experimental models of LPS-induced depression demonstrate high consistency among neuroinflammation, oxidative/nitrosative stress, mitochondrial dysfunction, and the development of depressive behaviors [11]. In this context, nitro-oxidative stress has been proposed as an important pathophysiological mechanism involved in mood disorders, since persistent ROS and RNS production promotes progressive neuronal damage, neurochemical alterations, and maintenance of chronic cerebral inflammation [154].

Therefore, current evidence demonstrates that LPS promotes activation of TLR4/NF-κB-dependent inflammatory pathways, increasing iNOS expression and favoring intense production of NO and superoxide anion. The interaction between these species leads to peroxynitrite formation and establishment of oxido-nitrosative stress in the central nervous system. Peroxynitrite amplifies neuroinflammation, intensifies mitochondrial dysfunction, and promotes neurochemical alterations associated with depression, representing an important molecular link between inflammation, cellular damage, and the development of depressive behaviors [67,116,149].

### 6.5. Imbalance of Antioxidant Defenses Caused by LPS

Antioxidant imbalance has been widely associated with the development of depression, although, by itself, it is not sufficient to trigger the disorder. Oxidative stress constitutes one of the main factors involved in the maintenance of neuroinflammation, neuronal damage, and other dysfunctions related to symptoms observed in depressive conditions [138].

In this context, LPS acts as a potent trigger of oxidative imbalance by promoting intense activation of the innate immune system. This imbalance occurs following the recognition of LPS by the TLR4/MD-2 complex present on the membrane of macrophages, microglia, and astrocytes, triggering activation of the Myeloid Differentiation Primary Response 88 (MyD88)-dependent pathway [24]. Initially, recruitment of the adaptor protein TIR domain-containing adaptor protein (TIRAP) occurs, which is responsible for mediating the interaction between TLR4 and MyD88 through the Toll/Interleukin-1 receptor (TIR) domain; subsequently, this interaction promotes recruitment and phosphorylation of interleukin-1 receptor-associated kinases (IRAKs), especially IRAK4 and IRAK1, culminating in activation of TNF receptor-associated factor 6 (TRAF6) [155]. Subsequently, TRAF6, in association with the enzymes Uev1A and Ubc13, promotes the formation of K63/M1 polyubiquitination chains, which function as a platform allowing recruitment and activation of the TAK1/TAB complex, ultimately leading to phosphorylation and nuclear translocation of NF-κB and AP1 factors. These factors promote a marked increase in the transcription of pro-inflammatory cytokines, including TNF-α, IL-1β, and IL-6, which amplify the local and systemic inflammatory response and directly stimulate cellular mechanisms associated with free radical production [24,156].

The production of these pro-inflammatory cytokines stimulates the activity of the enzyme indoleamine-2,3-dioxygenase (IDO), responsible for the degradation of tryptophan into kynurenine, in addition to increasing the activity of kynurenine-3-monooxygenase (KMO), favoring the metabolic routing of kynurenine toward the formation of neurotoxic metabolites, particularly quinolinic acid (QUIN) [138]. QUIN acts as an agonist of NMDA receptors (NMDA_R_), promoting neuronal glutamatergic hyperactivation, which generates an excessive increase in intracellular calcium (Ca^2+^) concentration, leading to mitochondrial calcium overload and depolarization of the inner mitochondrial membrane, thereby intensifying reactive oxygen species production. Studies have revealed a reciprocal interaction between oxidative stress and mitochondrial dysfunction through mitochondrial reactive oxygen species (mtROS), neuroinflammation, and excessive glutamate-induced Ca^2+^ influx [129,140].

In addition, another component related to this oxidative imbalance is the activation of the NADPH oxidase (NOX) enzyme, considered one of the major cellular sources of reactive oxygen species. Pro-inflammatory cytokines, particularly TNF-α, induce activation of this enzymatic complex in microglial cells, macrophages, and astrocytes through phosphorylation of cytosolic subunits of the NOX complex, especially p47phox, p67phox, and p40phox. Following phosphorylation, these subunits migrate from the cytosol to the plasma membrane, where they associate with the transmembrane subunits gp91phox (NOX2) and p22phox, forming the functional enzymatic complex. This complex promotes electron transfer from NADPH to molecular oxygen, generating superoxide anion, which can subsequently be converted into other reactive species, such as H2O2 and hydroxyl radicals, significantly increasing cellular oxidative damage [130,138].

In the central nervous system, this process is of great importance due to persistent microglial activation. Reactive oxygen species produced by microglial NOX act not only as cytotoxic molecules but also as intracellular second messengers capable of further amplifying activation of NF-κB and other pro-inflammatory pathways [130,138]. Thus, a positive feedback cycle is established between neuroinflammation and oxidative stress, contributing to synaptic damage, reduced hippocampal neurogenesis, impaired serotonergic and glutamatergic neurotransmission, and, consequently, neurobiological alterations associated with the pathophysiology of depression.

As a consequence, the persistent excess of reactive oxygen species associated with the progressive reduction in cellular antioxidant capacity favors the establishment of a chronic state of oxidative stress in the central nervous system. Under physiological conditions, antioxidant enzymes such as superoxide dismutase (SOD), catalase (CAT), glutathione peroxidase (GPx), and the reduced glutathione (GSH) system act by neutralizing free radicals and preserving cellular redox homeostasis [55]. However, prolonged exposure to LPS and the pro-inflammatory environment induced by TNF-α, IL-1β, and IL-6 impairs the activity of these antioxidant systems, mainly through functional inhibition of the nuclear factor erythroid 2-related factor 2 (Nrf2) pathway, considered one of the principal regulatory mechanisms of the endogenous antioxidant response [157]. Reduced Nrf2 activity decreases the transcription of antioxidant and cytoprotective genes, favoring the progressive accumulation of ROS and intensifying neuronal oxidative damage [138,156].

These neurobiological alterations directly affect brain regions involved in emotional regulation, such as the hippocampus, prefrontal cortex, and amygdala, contributing to behavioral manifestations associated with depression. Thus, LPS constitutes an important experimental model for understanding how the interaction among neuroinflammation, mitochondrial dysfunction, and imbalance between pro-oxidant and antioxidant systems participates in the pathophysiology of depression.

### 6.6. The Role of Nrf2

In addition to the persistent increase in ROS production, another component that favors this imbalance is the reduction in Nrf2 levels induced by LPS. Considered one of the main regulators of cellular redox homeostasis, Nrf2 controls the transcription of several antioxidant and cytoprotective genes responsible for neutralizing free radicals and maintaining cellular integrity [158].

Under physiological conditions, Nrf2 remains associated with Kelch-like ECH-associated protein 1 (Keap1) in the cytoplasm, where it undergoes basal proteasomal degradation. However, under conditions of moderate stress, dissociation of the Nrf2/Keap1 complex occurs, allowing nuclear translocation of Nrf2 and subsequent activation of Antioxidant Response Elements (ARE), promoting increased expression of antioxidant enzymes such as heme oxygenase-1 (HO-1), NAD(P)H quinone oxidoreductase 1 (NQO1), superoxide dismutase (SOD), catalase (CAT), and glutathione peroxidase (GPx) [158].

Whereas low or initial levels of stress and neuroinflammation may activate Nrf2 as a compensatory response of the organism to combat oxidative imbalance, excessive nitrosative and oxidative stress induced by high doses of LPS (or prolonged exposure) leads to Nrf2 downregulation and degradation [159]. One of the principal mechanisms involved consists of suppression of silent information regulator 1 (SIRT1), an NAD^+^-dependent deacetylase responsible for regulation of redox homeostasis and Nrf2 stability. Under physiological conditions, SIRT1 favors the Nrf2-mediated antioxidant pathway through deacetylation of proteins associated with the antioxidant response and indirect inhibition of NF-κB-dependent pro-inflammatory signaling [160].

However, studies demonstrate that LPS exposure significantly reduces SIRT1 expression and activity, impairing Nrf2 activation and exacerbating cellular oxidative stress [161]. In parallel, reduced SIRT1 activity favors increased acetylation of the NF-κB RelA/p65 subunit, potentiating its transcriptional activity and promoting greater expression of pro-inflammatory cytokines such as TNF-α, IL-1β, and IL-6, thereby amplifying the neuroinflammatory and pro-oxidative environment [161,162].

Furthermore, another mechanism associated with LPS-induced reduction in Nrf2 expression/activation is based on persistent activation of glycogen synthase kinase-3 beta (GSK3β), which constitutes another important mechanism of negative regulation of this factor. GSK3β induces phosphorylation of Nrf2 at specific residues, allowing ubiquitin ligase β-transducin repeat-containing protein (β-TrCP) to target it for proteasomal degradation independently of Keap1 [159]. In parallel, hyperactivation of the TLR4/NF-κB pathway establishes an antagonistic relationship with Nrf2. NF-κB competes with Nrf2 for the coactivator CREB-binding protein (CBP), which, upon binding, prevents this factor from initiating transcription of antioxidant genes, favoring oxidative imbalance [156,161]. Thus, the functional loss of Nrf2 removes an important negative regulatory mechanism of inflammation, favoring persistent neuroinflammation and progressive oxidative damage.

**Figure 5:**
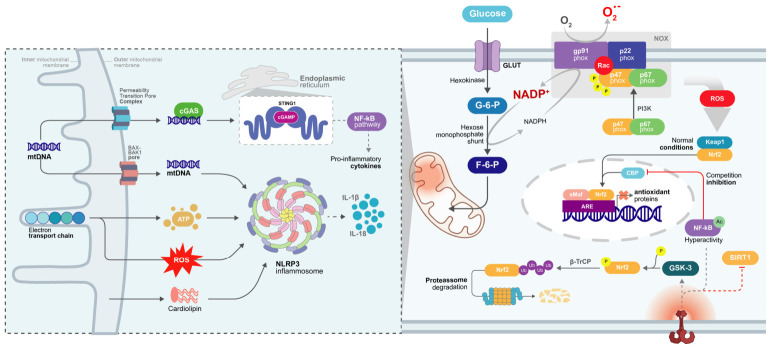
Mitochondrial dysfunction and oxidative stress pathways involved in LPS-induced neuroinflammation and depression. The figure illustrates interconnected mechanisms linking mitochondrial dysfunction, oxidative stress, and neuroinflammatory signaling associated with LPS-induced depression. Mitochondrial damage promotes the release of mitochondrial DNA (mtDNA), ATP, cardiolipin, and excessive reactive oxygen species (ROS), which contribute to activation of the NLRP3 inflammasome and release of pro-inflammatory cytokines, including IL-1β and IL-18. Cytosolic mtDNA also activates the cGAS–STING signaling pathway, leading to NF-κB activation and amplification of inflammatory responses. Simultaneously, glucose metabolism through the hexose monophosphate pathway generates NADPH, which fuels NADPH oxidase (NOX) activation and further ROS production. Excessive ROS generation disrupts antioxidant homeostasis by suppressing the Nrf2 pathway through Keap1, GSK-3β, NF-κB, and SIRT1-dependent mechanisms, impairing transcription of antioxidant proteins and promoting proteasomal degradation of Nrf2. Together, these mechanisms establish a self-perpetuating cycle of oxidative stress, mitochondrial impairment, and persistent neuroinflammation that may contribute to the pathophysiology of depression. Arrows without points indicate negative modulation of the target. The “x” symbol above the arrows indicates direct inhibition of the formation of pathway-related products.

As a final consequence of these mechanisms, a significant reduction occurs in the expression of antioxidant enzymes regulated by Nrf2, including HO-1, NQO1, SOD, catalase, and glutathione peroxidase, in addition to the progressive depletion of reduced glutathione levels [55,138]. The loss of these antioxidant defenses favors the exacerbated accumulation of reactive oxygen and nitrogen species, promoting lipid peroxidation and mitochondrial damage. These alterations directly contribute to the maintenance of neuroinflammation and to the neurobiological alterations associated with the pathophysiology of depression (Figure 5).

## 7. Experimental Models for Studying Xenobiotic Effects in Neuroinflammation

Experimental models constitute fundamental tools for investigating neuroimmune responses to injury, infections, and exposure to xenobiotics. These models encompass different levels of complexity, ranging from systems based on cell cultures to more integrative approaches, such as animal models and platforms that mimic functional tissue characteristics (organoids), allowing the analysis of multiple parameters involved in neuroinflammation [163,164,165,166,167]. In this context, systemic inflammation models based on LPS administration are widely used because they reproduce depression-like behaviors associated with neurochemical, neuroimmune, and specific brain circuit alterations, particularly in the prefrontal cortex, amygdala, striatum, and hippocampus [163,168,169].

Different LPS administration strategies—including variations in dose, regimen, timing of exposure, and animal strain—significantly influence the observed outcomes. Acute or short-term protocols tend to induce transient responses resembling a sickness-like state, whereas repeated exposures or high doses are associated with the development of anhedonia and other depression-related behaviors, such as exploratory capacity, behavioral despair and immobility [169,170,171,172]. In addition, these experimental variations modulate different neurobiological pathways, including increased pro-inflammatory cytokines, activation of indoleamine 2,3-dioxygenase (IDO) and NF-κB, microglial activation, dysregulation of the hypothalamic–pituitary–adrenal axis, and reduced hippocampal neurogenesis [170,171,173,174].

In this regard, LPS-induced in vivo models present several methodological advantages. Their administration is considered technically simple and reproducible and can be applied in different animal species, such as rodents and fish [148,175,176]. This model promotes a systemic inflammatory phenotype characterized by rapid elevations of pro-inflammatory cytokines (TNF-α, IL-1β, and IL-6), in addition to physiological dysfunctions and oxidative stress [176,177]. In parallel, it induces depression-like behaviors associated with increased inflammatory mediators at central and peripheral levels, allowing the correlation between behavioral alterations and activation of the immune system [146,172,178]. To provide a clearer methodological overview of these experimental approaches, Table 2 summarizes the main LPS-induced depression protocols used in rodents, including species, administration regimens, behavioral assessment timelines, and corresponding references.

Nevertheless, LPS-induced inflammation models present important limitations. Among these, the difficulty in distinguishing depression-like behaviors from adaptive responses to acute inflammatory conditions (sickness behavior) should be highlighted, which may lead to misinterpretation of behavioral outcomes [172,184]. In addition, structural variations in LPS derived from different bacterial species may result in distinct inflammatory activation profiles, affecting the reproducibility and sensitivity of bioassays [185,186]. From a translational perspective, interspecies differences in xenobiotic metabolism represent an additional challenge. Animal species exhibit significant variations in enzymatic machinery, including cytochrome P450 isoforms such as CYP1A, CYP2C, CYP2D, and CYP3A, which limits the direct extrapolation of pharmacokinetic data to humans [187,188]. Similarly, other enzymes involved in metabolism, such as aldehyde oxidase, also exhibit relevant interspecies differences, contributing to distinct biotransformation and toxicity profiles [188].

Regarding in vitro neuroinflammation models, these range from simpler systems, such as microglial cell lines, to more complex approaches, including tricultures, systems based on induced pluripotent stem cells (iPSCs), organoids, and organotypic slices [165,166,189,190]. Monocultures are widely used in screening and signaling pathway investigations, with cell lines such as BV2 and MMC being particularly employed in high-throughput mechanistic analyses involving LPS/TLR [189]. In contrast, primary microglial cultures exhibit greater similarity to the in vivo environment, although they are limited by lower experimental yield [191].

Coculture models, especially those involving microglia and neurons, allow the evaluation of relevant cellular interactions, such as the induction of neuronal death mediated by microglial activation through LPS/IFN-γ, in addition to enabling the analysis of the neuroprotective potential of anti-inflammatory compounds [192,193]. In a more integrated manner, triculture systems composed of neurons, astrocytes, and microglia exhibit greater predictive capacity for in vivo neuroinflammation and neurotoxicity events, including specific responses such as LPS-induced astrocytic hypertrophy, which is absent in more simplified models [190,194,195].

Additionally, organotypic brain slices preserve tissue cytoarchitecture and maintain microglial transcriptional characteristics similar to those observed in adult individuals, constituting a relevant alternative for evaluating modulators of neuroimmune activation in a tissue context [165,196]. Finally, iPSC-derived models allow the investigation of processes such as cytokine secretion, cellular migration, and inflammatory interactions in controlled microenvironments, contributing to the mechanistic study of responses to stimuli such as LPS, TNF-α, and IL-1β [197]. Collectively, these in vitro models are widely used in elucidating molecular mechanisms of neuroinflammation, as well as in the initial screening of compounds with modulatory potential, enabling the identification of candidates with anti-inflammatory and neuroprotective properties. Representative in vitro LPS-based neuroinflammation models that reproduce cellular and molecular mechanisms associated with depression-like phenotypes are summarized in Table 3, including cellular systems, stimulation conditions, and main experimental readouts.

In this context, in vivo models allow the analysis of integrated organismal responses, whereas in vitro models enable the detailed investigation of cellular signaling pathways, in addition to allowing higher-throughput assays [164,194,200]. Animal models provide a systemic and physiologically relevant context, including the presence of the blood–brain barrier, systemic metabolism, aging, and interactions with peripheral immune cells. In neurotoxicity models, such as those based on LPS or neurotoxins, it is possible to observe not only neuroinflammatory processes but also alterations in neurotransmission and neurodegenerative events, which are particularly relevant for conditions such as Alzheimer’s disease and Parkinson’s disease [164,200].

However, in vivo approaches present important limitations, including ethical concerns, high costs, and interspecies differences in metabolism, signaling pathways, and brain organization, which hinder the extrapolation of findings to humans [201].On the other hand, in vitro models allow precise control of experimental variables, such as dose, environment, and exposure time to xenobiotics, and are widely used in toxicological studies and compound screening at molecular and cellular levels [194,202]. However, these systems lack a complete physiological microenvironment and present limitations in modeling chronic processes. Even more advanced approaches, such as tricultures, iPSC-derived systems, organoids, and blood–brain barrier models, although closer to the human context, still face challenges related to technical complexity, standardization, and cost [166,201,202,203]. Collectively, these approaches should be considered complementary, and integration between in vivo and in vitro models is essential for a broader understanding of the effects of xenobiotics on neuroinflammation.

In parallel, in silico methodologies support neuroinflammation research by evaluating the ability of compounds to cross the blood–brain barrier and reach the brain, metabolism, toxicity profiles, and possible neural or immunological interaction targets through tools such as Quantitative Structure–Activity Relationship (QSAR), molecular dynamics studies, docking, and network toxicology, increasingly being associated with in vitro and in vivo methods [204,205,206,207].

In this regard, machine learning pharmacophore models and hierarchical SVR may demonstrate probable blood–brain barrier permeability, as well as the proportion of free protein between plasma and brain, considering active transport and passive diffusion [204,206,208]. It is also possible to correlate dose exposure through the combination of QSAR with physiologically based pharmacokinetics (PBPK) in order to estimate CNS concentrations and classify compounds regarding neurotoxicity [204,206]. Regarding CYP-mediated metabolism, metabolism prediction can be performed through target- and ligand-based models, as well as prediction of toxic metabolite formation [207]. In addition, electrophilicity models based on Hard and Soft Acids and Bases (HSAB) detect potential neurotoxicity risks of new molecules with specificity and good predictive capability [209]. In this context, integrated QSAR/read-across systems have been proposed for the evaluation of neurotoxicity during development and adulthood [204].

Furthermore, studies use combined methods, including 2D/3D QSAR, molecular dynamics (MD), and docking to prioritize ligands for neurological targets such as NMDA and TNF-α, with the aim of analyzing excitotoxicity and modulation of neuroinflammation and synaptic function [205,210,211,212]. Regarding research on per- and polyfluoroalkyl substances (PFAS), combinations of methods—including Kyoto Encyclopedia of Genes and Genomes (KEGG) pathway analysis, prediction of absorption, distribution, metabolism, and toxicity (ADMET), molecular docking and dynamics, and target-network analysis—are used with the aim of identifying stable toxin–protein complexes and neurotoxic pathways [213].

Given the above, integration of in silico, in vitro, and in vivo approaches provides a more robust evaluation of the effects of xenobiotics on neuroinflammation. While in silico methods enable prediction of pharmacokinetic properties, exposure variability, and interactions with molecular targets, in vitro models contribute to elucidation of cellular mechanisms and initial compound screening. In turn, in vivo approaches provide evidence at the systemic level, including behavioral and functional aspects. Collectively, this integration improves predictive capacity, reduces dependence on animal models, and promotes the identification of relevant therapeutic targets.

## 8. Translational Implications for Drug Development

Collectively, the mechanisms discussed throughout this review indicate that LPS-induced neuroinflammation emerges from a complex interplay among innate immune activation, microglial signaling, oxidative stress, mitochondrial dysfunction, inflammasome activation, and neurotransmitter disturbances. These interconnected pathways not only contribute to the pathophysiology of inflammation-associated depression but also provide multiple molecular targets for therapeutic intervention. Experimental findings obtained from LPS-induced models have significantly advanced our understanding of inflammatory signaling pathways involved in depressive-like behavior, particularly mechanisms involving TLR4 activation, NLRP3 inflammasome signaling, oxidative stress amplification, and neuroimmune dysfunction [24,72,214]. However, despite substantial advances in mechanistic understanding, translating these findings into clinically effective therapeutic strategies remains a major challenge.

Although LPS-induced models successfully reproduce several neuroinflammatory and behavioral characteristics associated with depression, important limitations remain regarding their predictive capacity for human diseases. Firstly, in vivo studies only reflect “depressive-like” behavior, given that depressive disorders involve a subjective and individual component. Thus, the depressive genesis, which involves diverse factors such as psychosocial, genetic, and environmental factors, cannot be completely mimicked in the LPS-induced neuroinflammation model. Furthermore, experimental paradigms often represent acute or exaggerated inflammatory states that may not fully reflect the multifactorial and heterogeneous nature of depressive disorders observed in clinical settings [136]. In addition, substantial differences between species in neuroanatomical organization, immune responses, inflammatory sensitivity, and receptor expression profiles can significantly influence translational results [11]. Behavioral paradigms commonly employed in LPS models, including the forced swimming test, tail suspension test, and sucrose preference test, may also partially overlap with sickness behavior rather than exclusively reflecting depressive symptomatology, creating additional interpretative limitations [215]. Similarly, although advanced in vitro platforms such as multicellular cocultures, microfluidic systems, and organoid-based models have improved experimental complexity, challenges involving reproducibility, standardization, vascularization, and representation of systemic immune interactions remain unresolved [166,201].

Importantly, mechanistic pathways identified through LPS-based experimental systems have revealed several potential therapeutic targets relevant to neuroinflammation-associated depression. Among these, TLR4 signaling represents an upstream pathway capable of initiating inflammatory amplification through MyD88- and TRIF-dependent cascades, ultimately leading to NF-κB activation and cytokine production [24]. Similarly, increasing evidence supports the NLRP3 inflammasome as a critical molecular hub linking oxidative stress to inflammatory amplification. Excessive mitochondrial ROS generation, TXNIP activation, potassium efflux, and mitochondrial dysfunction collectively contribute to NLRP3 assembly and downstream activation of caspase-1, IL-1β, IL-18, and pyroptotic signaling. Consequently, therapeutic strategies targeting inflammasome activation, ASC recruitment, caspase-1 signaling, or pyroptosis-related pathways have emerged as promising alternatives for limiting persistent neuroinflammatory responses [24,90,216,217].

Oxidative stress pathways have also become attractive therapeutic targets because excessive ROS and reactive nitrogen species generation contributes directly to neuronal dysfunction and inflammatory persistence. In particular, impairment of endogenous antioxidant responses involving Nrf2 signaling has been consistently associated with LPS-induced neuroinflammation [161]. Suppression of Nrf2 activity through mechanisms involving SIRT1 downregulation, persistent NF-κB activation, and GSK3β-mediated degradation favors sustained oxidative imbalance and amplification of inflammatory signaling [59,159,161]. Therefore, compounds capable of restoring redox homeostasis or simultaneously modulating inflammatory and oxidative pathways may offer important therapeutic ad-vantages over single-target interventions.

Despite promising preclinical evidence, several xenobiotic-related factors may com-promise successful translation into clinical applications. Drug efficacy observed under experimental conditions does not necessarily predict therapeutic performance in humans because central nervous system exposure is strongly influenced by blood–brain barrier permeability, tissue distribution, transporter activity, systemic clearance, and metabolic stability [218,219]. Additionally, interspecies differences in cytochrome P450 activity, xenobiotic metabolism, and target engagement may contribute substantially to discrepancies between preclinical and clinical findings [188]. Such factors reinforce the importance of integrating mechanistic evidence with pharmacokinetic and toxicological considerations during therapeutic development.

Recent advances in computational and integrative approaches may help overcome some of these translational limitations. In silico methodologies, including quantitative structure–activity relationship (QSAR) models, machine learning algorithms, molecular docking, physiologically based pharmacokinetic/pharmacodynamic (PBPK-PD) approaches, and blood–brain barrier prediction tools have increasingly contributed to neuropharmacological research [220,221]. These approaches facilitate prediction of target engagement, estimation of CNS penetration, optimization of molecular properties, and identification of compounds with improved translational potential. Therefore, combining experimental evidence with computational strategies may improve prediction accuracy and accelerate the development of more effective therapeutic interventions targeting neuroinflammation-associated depression.

## 9. Conclusions

Although considerable progress has been made in elucidating these molecular pathways, important challenges remain. Most evidence is derived from acute preclinical models that only partially reproduce the complexity and heterogeneity of major depressive disorder in humans. Consequently, greater methodological standardization and the validation of translational biomarkers are needed to improve the clinical relevance of experimental findings.

Future research should focus on integrating molecular, cellular, and behavioral data to better characterize inflammation-associated depression and identify reliable therapeutic targets. In particular, multitarget approaches aimed at simultaneously modulating oxidative stress, mitochondrial dysfunction, and inflammasome activation may represent promising strategies for improving treatment outcomes and facilitating the translation of preclinical discoveries into clinical practice.

## Figures and Tables

**Table 1:** Comparison of the major signaling pathways involved in LPS-induced depression, their principal molecular targets, representative therapeutic compounds or pharmacological strategies, main pharmacological effects, and key references discussed in this review.

Signaling Pathway	Principal Molecular Targets	Representative Therapeutic Compounds	Main Pharmacological Effects	Ref.
*TLR4/MyD88/NF-κB*	TLR4, MyD88, NF-κB	Gypenosides	Suppression of NF-κB activation, inhibition of M1 microglial polarization, reduction in TNF-α, IL-1β and IL-6 production	[45,46,52]
*MAPK (ERK/JNK/p38)*	ERK1/2, JNK, p38 MAPK	Punicalagin; ω-3 DPA	Inhibition of MAPK signaling (ERK/JNK/p38 or p38), attenuation of oxidative stress, suppression of neuroinflammation, reduction in pro-inflammatory cytokine production, and promotion of neuroprotection	[47,49,53,54,55]
*PI3K/AKT/FOXO1*	PI3K, AKT, FOXO1, TLR4	Kaixin Jieyu Granule	Activation of PI3K/AKT signaling, inhibition of FOXO1-mediated TLR4 expression, suppression of neuroinflammation, reduction in TNF-α, IL-1β and IL-6 production, and attenuation of depression-like behavior	[56]
*JAK1/STAT3–CREB/BDNF*	JAK1, STAT3, CREB, BDNF	Echinacoside	Activation of JAK1/STAT3 and CREB/BDNF signaling, promotion of microglial M2 polarization, suppression of neuroinflammation, enhancement of hippocampal neurogenesis and neuronal plasticity, and antidepressant-like effects	[57]
*Monoaminergic signaling*	SERT, NF-κB, MAPK (ERK, JNK, p38), NLRP3 inflammasome, iNOS, COX-2, IL-1β, IL-6, TNF-α	Fluoxetine	Suppression of microglial activation, inhibition of NF-κB/MAPK signaling, reduction in pro-inflammatory cytokines and nitric oxide production, attenuation of oxidative stress, and antidepressant-like effects	[62]
*NADPH oxidase (NOX2)/ROS*	NOX2, NADPH, ROS	Exogenous NADPH	Suppression of microglial activation, attenuation of oxidative stress, reduction in IL-1β, TNF-α and IFN-γ production, preservation of synaptic and myelin integrity, and alleviation of depression-like behaviors	[63,64,65,66]
*Nitrosative stress*	iNOS, NO, ONOO^−^, PPAR-α	Honokiol; WY-14643	Attenuation of iNOS-derived nitric oxide and peroxynitrite formation, reduction in oxidative and nitrosative stress, suppression of neuroinflammatory cytokine production, restoration of endogenous antioxidant defenses, and improvement of depression-like behaviors	[67,68,69]
*NLRP3 inflammasome*	NLRP3, TXNIP, NEK7, ASC, Caspase-1, Gasdermin D, P2X7	CY-09;	Inhibition of NLRP3 inflammasome assembly and activation, suppression of Caspase-1/Gasdermin D-mediated pyroptosis, reduction in IL-1β and IL-18 release, attenuation of chronic neuroinflammation, and improvement of depression-like behaviors	[58,61,70,71,72]
*Mitochondrial dysfunction*	mtROS, PGC-1α, mitochondrial membrane potential (ΔΨm), cardiolipin	Resveratrol;	Reduction in mitochondrial ROS production, restoration of mitochondrial bioenergetics and membrane potential, attenuation of neuroinflammation, and improvement of depression-like behaviors	[73,74,75,76,77,78,79,80,81]

**Table 2:** Experimental protocols of lipopolysaccharide (LPS)-induced depression in rodents. The table summarizes different experimental LPS administration regimens used to induce depression-like behaviors in mice and rats, including acute, subchronic/chronic, central, stress-combined, and Parkinson’s disease-like condition-associated models. The species/strain, administration protocol, behavioral assessment time, and corresponding references are presented. Abbreviations: LPS, lipopolysaccharide; i.p., intraperitoneal; FST, *forced swimming test*; TST, *tail suspension test*; SPT, *sucrose preference test*; OFT, *open field test*; EPM, *elevated plus maze*; DRN, *dorsal raphe nucleus*; dPD, *depression in Parkinson’s disease-like condition*; MRI, *magnetic resonance imaging*; c-Fos, neuronal activation marker; Iba-1, *ionized calcium-binding adapter molecule 1*; TH, tyrosine hydroxylase.

Experimental Model/Objective	Species/Strain	LPS Protocol	Assessment and Timeline	Ref.
General synthesis of acute LPS models	Mice of different strains	0.5–1 mg/kg, i.p., single dose; protocols with 0.8 or 0.83 mg/kg	FST, TST, and/or SPT generally approximately 24 h after administration; in some protocols, between 4 and 28 h	[11,170,179,180]
Single-dose model with prolonged effect	Adolescent ddY mice	1.66 mg/kg, i.p., compared with 0.83 mg/kg, both administered as a single dose	Depression-like behavior observed for up to 14 days, compared with approximately 4 days for 0.83 mg/kg	[170]
Acute/subacute model	C57BL/6 mice	i.p. administration for one week (dose not specified in the methodology)	Behavioral assessment at the end of week 1: OFT, TST, FST, and SPT. The model reduced exploration in the OFT, increased immobility in the FST, and reduced sucrose preference in the SPT; TST showed no significant difference at this stage	[181]
Subchronic protocol with dose escalation	C57BL/6J mice	Weeks 1–2: 0.208, 0.415, and 0.83 mg/kg, i.p.; weeks 3–4: 0.208, 0.415, 0.83, and 0.83 mg/kg, i.p.	Behavioral assessments performed at the end of each week, including OFT, TST, FST, and SPT according to the experimental period. The objective was to induce persistent inflammation and longer-lasting depression-like behavior	[181]
Acute/subacute central model through LPS microinfusion into the dorsal raphe nucleus (DRN)	C57BL/6 mice	LPS microinfusion into the DRN for 1 week. Volume/concentration: 5 µL LPS at 0.5 mg/mL or 2.5 µL LPS at 1 mg/mL	Behavioral assessment after the microinfusion period, including OFT, TST, and/or FST, in addition to formaldehyde and cytokine analyses in the midbrain. The objective was to evaluate whether DRN LPS administration induced local neuroinflammation and depression-like behavior	[181]
Acute model	C57BL/6J mice	Single dose of 0.83 mg/kg i.p.	Behavioral tests performed 24 h after injection. FST, TST, OFT, and EPM were used	[182]
Short subchronic/chronic model	C57BL/6J mice	0.5 mg/kg/day i.p. for 7 days	Behavioral tests performed 24 h after the last injection on day 7. FST, TST, OFT, and EPM were used	[182]
LPS model compared with maternal separation	C57BL/6 mice	2 mg/kg i.p. for 5 consecutive days	Behavioral tests were performed after the administration period in the following order: OFT, EPM, FST, and TST. Two tests were conducted per day	[146]
LPS-induced dPD model—1 or 2 days	Sprague Dawley rats	0.5 mg/kg, i.p., for 1 day or for two days	Assessments performed 24 h after the last injection: SPT, OFT, and rotarod. Immunohistochemical analyses of c-Fos, Iba-1, and TH, as well as hippocampal cytokine analyses, were also performed	[169]
LPS-induced dPD model—4 days	Sprague Dawley rats	0.5 mg/kg, i.p., for 4 consecutive days	Assessments performed 24 h after the last injection: SPT, OFT, rotarod, and MRI; c-Fos, Iba-1, TH, and cytokine analyses were performed after behavioral tests	[169]
Combined depression model induced by chronic restraint stress + LPS	Mice of different strains, including ICR, C57BL/6J, and BALB/c	1 mg/kg i.p., once daily for 7 consecutive days, followed by chronic restraint stress beginning 30 min after injection	Depression-like behaviors were evaluated following the 7-day combined protocol	[172]
LPS-induced depression model with assessment of neuroinflammation, oxidative stress, and peroxiredoxins	ICR mice	1 mg/kg/day, i.p., 0.1 mL/day, for 7 consecutive days	Behavioral tests 24 h after the last injection: OFT, SPT, and FST; serum TNF-α, IL-1β, and TGF-β1 levels measured by ELISA; evaluation of Prdx1, Prdx2, Prdx4, and Prdx5 in the hippocampus by IHC, WB, and RT-qPCR	[183]
LPS-induced depressive state associated with neuroinflammation and differential assessment of the hippocampus and prefrontal cortex	Wild-type/house mice	1 mg/kg, i.p.; two administration phases with three injections per phase and a 7-day interval between phases	Phase 1: OFT, TST, SPT, and Y-maze; tests performed on day 3, 4 h after the third injection, except SPT, which was performed after the second injection for a 24 h duration. Phase 2: hippocampus, prefrontal cortex, and spleen collected 4 h after the third injection for PCR, histological, and immunohistochemical analyses	[168]

**Table 3:** In vitro neuroinflammation protocols based on lipopolysaccharide (LPS) that mimic mechanisms associated with LPS-induced depression models. The table summarizes cellular systems, LPS stimulation conditions, and the main readouts used to evaluate microglial/astrocytic activation, cytokine release, oxidative stress, and neuronal injury. Abbreviations: LPS, lipopolysaccharide; BV2, murine microglial cell line; RAECs, rat aortic endothelial cells; CMHE, conditioned medium from a hypertensive environment; iPSC, induced pluripotent stem cells; DIV, day in vitro; IL, interleukin; IL1B, interleukin-1 beta encoding gene; TNF, tumor necrosis factor encoding gene; TNF-α, tumor necrosis factor-alpha; TGF-β1, transforming growth factor beta 1; ROS, reactive oxygen species; NO, nitric oxide; Prdxs, peroxiredoxins; ELISA, enzyme-linked immunosorbent assay; WB, Western blot; RT-qPCR, reverse transcription quantitative polymerase chain reaction; FGF2, fibroblast growth factor 2; Iba1, ionized calcium-binding adapter molecule 1; GFAP, glial fibrillary acidic protein; ET-1, endothelin-1; MCP-1, monocyte chemoattractant protein 1; VCAM-1, vascular cell adhesion molecule 1; NE, norepinephrine; DA, dopamine; 5-HT, serotonin; TLR4, Toll-like receptor 4; NF-κB, nuclear factor kappa B; C3, complement component 3; Ca^2+^, calcium ion; ↑, increase; ↓, reduction.

Cellular System/Context	Experimental Model/Objective	LPS Conditions	Main Readouts/Outcomes	Ref.
BV2 microglia	In vitro model of microglial activation associated with neuroinflammatory mechanisms of LPS-induced depression	LPS 1 µg/mL for 24 h	IL-1β, TNF-α, TGF-β1, ROS, NO, and Prdxs assessed by ELISA, fluorescence, WB/RT-qPCR	[183]
Primary mouse hippocampal glial cultures	LPS-induced neuroinflammation model with evaluation of the effect of FGF2 on the microglial phenotype	LPS 100 ng/mL added to the medium for 6 or 12 h, alone or combined with FGF2 200 ng/mL	Immunocytochemistry for Iba1 and GFAP; microglial area; proportion of ramified and amoeboid microglial phenotypes. LPS induced a shift toward an amoeboid/pro-inflammatory phenotype; FGF2 reduced microglial area and promoted restoration of the ramified phenotype	[168]
RAECs → hippocampal neuron–cortical microglia triculture	Endothelial-neuroglial inflammation model associated with corticosterone	RAECs exposed to LPS 1 µg/mL for 24 h; conditioned supernatant (CMHE, 10%) combined with corticosterone 200 µM and applied to neuron–microglia coculture for 24 h	NO ↓, ET-1 ↑, MCP-1 ↑, VCAM-1 ↑, TNF-α ↑, and IL-1β ↑. In the triculture: neuronal viability ↓, NE/DA/5-HT ↓, ROS ↑, apoptosis ↑, pro-inflammatory microglial polarization, and TLR4/NF-κB activation	[198]
Rat cortical triculture: neurons–astrocytes–microglia	Multicellular neuroinflammatory response to LPS	LPS 5 µg/mL for 48 h, applied on day 7 in vitro (DIV 7)	Caspase-3/7 ↑, astrocytic hypertrophy ↑, microglial area ↑, TNF-α ↑, IL-1α ↑, IL-1β ↑, and IL-6 ↑; absent or minimal response in cocultures without microglia	[194]
Human iPSC-derived microglia–astrocyte coculture	Conventional model and microfluidic platform for studying glial interaction in neuroinflammation	LPS 100 ng/mL for 24 h in monocultures and cocultures; comparison among microglia, astrocytes, and microglia–astrocyte cocultures	In microglia: CXCL5 ↑, CCL2 ↑, CXCL8 ↑, IL-6 ↑, IL-10 ↑, TNF-α ↑, and IL-1β ↑; in coculture: modulation of the inflammatory response, microglial migration, phagocytosis, and C3	[197]
Human iPSC triculture: neurons–astrocytes–microglia	Microglia–astrocyte–neuron inflammatory cascade model	LPS 100 ng/mL after 3–4 weeks of culture; evaluations at 0, 0.5, 1, 3, 6, and 24 h; protein quantification after 24 h	Microglial morphology; TNF ↑ and IL1B ↑; TNF-α ↑ and IL-1β ↑; nuclear translocation of NF-κB in microglia followed by astrocytes; neuronal excitability ↑ assessed by Ca^2+^ imaging	[199]

## Data Availability

No new data were created or analyzed in this study.

## Abbreviations

The following abbreviations are used in this manuscript:

5-HT	Serotonin
ADMET	Absorption, Distribution, Metabolism, Excretion, and Toxicity
AI	Artificial Intelligence
ASC	Apoptosis-associated speck-like protein containing a CARD
ATP	Adenosine triphosphate
BBB	Blood–Brain Barrier
BDNF	Brain-Derived Neurotrophic Factor
BV2	Murine microglial cell line
C3	Complement component 3
Ca^2+^	Calcium ion
CARD	Caspase recruitment domain
CASP-1	Caspase-1
CCL2	C-C motif chemokine ligand 2
c-Fos	Cellular Fos proto-oncogene (neuronal activation marker)
cGAS	Cyclic GMP–AMP synthase
CMHE	Conditioned Medium from a Hypertensive Environment
CNS	Central Nervous System
COX-2	Cyclooxygenase-2
CREB	cAMP Response Element-Binding Protein
CXCL5	C-X-C motif chemokine ligand 5
CXCL8	C-X-C motif chemokine ligand 8
DA	Dopamine
DAMPs	Damage-Associated Molecular Patterns
DIV	Day In Vitro
dPD	Depression in Parkinson’s Disease-like condition
DRN	Dorsal Raphe Nucleus
DRP1	Dynamin-Related Protein 1
ELISA	Enzyme-Linked Immunosorbent Assay
EPM	Elevated Plus Maze
ERK1/2	Extracellular Signal-Regulated Kinases 1/2
ET-1	Endothelin-1
FGF2	Fibroblast Growth Factor 2
FST	Forced Swimming Test
GFAP	Glial Fibrillary Acidic Protein
GPI	Glycosylphosphatidylinositol
GSDMD	Gasdermin D
GSH	Glutathione
GSK-3β	Glycogen Synthase Kinase-3 Beta
HMGB1	High Mobility Group Box 1
HSAB	Hard and Soft Acids and Bases
Iba1 (or Iba-1)	Ionized Calcium-Binding Adapter Molecule 1
ICR	Institute of Cancer Research (mouse strain)
IDO	Indoleamine 2,3-Dioxygenase
IFN-γ	Interferon Gamma
IL	Interleukin
IL-1α	Interleukin-1 Alpha
IL-1β	Interleukin-1 Beta
IL-6	Interleukin-6
IL-10	Interleukin-10
IL-18	Interleukin-18
IL1B	Interleukin-1 Beta Encoding Gene
iNOS	Inducible Nitric Oxide Synthase
i.p.	Intraperitoneal
iPSC	Induced Pluripotent Stem Cell
IRAK	Interleukin-1 Receptor-Associated Kinase
KEAP1	Kelch-like ECH-Associated Protein 1
KEGG	Kyoto Encyclopedia of Genes and Genomes
LBP	LPS-Binding Protein
LPS	Lipopolysaccharide
LRR	Leucine-Rich Repeat
MAVS	Mitochondrial Antiviral Signaling Protein
MCP-1	Monocyte Chemoattractant Protein-1
MDA	Malondialdehyde
MD	Molecular Dynamics
MD-2	Myeloid Differentiation Protein-2
Mfn1	Mitofusin-1
Mfn2	Mitofusin-2
MMC	Mixed Microglial Cell culture (or microglial cell line, conforme definido no manuscrito)
MRI	Magnetic Resonance Imaging
mtDNA	Mitochondrial DNA
MyD88	Myeloid Differentiation Primary Response Protein 88
NE	Norepinephrine
NEK7	NIMA-Related Kinase 7
NF-κB	Nuclear Factor Kappa B
NLRP3	NOD-, LRR-, and Pyrin Domain-Containing Protein 3
NMDAR	N-Methyl-D-Aspartate Receptor
NO	Nitric Oxide
NOX	NADPH Oxidase
NOX2	NADPH Oxidase 2
NRF1	Nuclear Respiratory Factor 1
Nrf2	Nuclear Factor Erythroid 2-Related Factor 2
OFT	Open Field Test
PBPK	Physiologically Based Pharmacokinetics
PFAS	Per- and Polyfluoroalkyl Substances
PGC-1α	Peroxisome Proliferator-Activated Receptor Gamma Coactivator-1 Alpha
PI3K	Phosphoinositide 3-Kinase
PK/PD	Pharmacokinetic/Pharmacodynamic
PPAR-α	Peroxisome Proliferator-Activated Receptor Alpha
Prdxs	Peroxiredoxins
P2X7	Purinergic Receptor P2X7
QSAR	Quantitative Structure–Activity Relationship
RAECs	Rat Aortic Endothelial Cells
RNS	Reactive Nitrogen Species
ROS	Reactive Oxygen Species
RT-qPCR	Reverse Transcription Quantitative Polymerase Chain Reaction
SIRT1	Sirtuin 1
SOD	Superoxide Dismutase
SPT	Sucrose Preference Test
STING	Stimulator of Interferon Genes
SVR	Support Vector Regression
Tfam	Mitochondrial Transcription Factor A
TGF-β1	Transforming Growth Factor Beta 1
TH	Tyrosine Hydroxylase
TIR	Toll/Interleukin-1 Receptor
TLR4	Toll-Like Receptor 4
TNF	Tumor Necrosis Factor Encoding Gene
TNF-α	Tumor Necrosis Factor Alpha
TRAF6	TNF Receptor-Associated Factor 6
TrkB	Tropomyosin Receptor Kinase B
TST	Tail Suspension Test
TXNIP	Thioredoxin-Interacting Protein
VCAM-1	Vascular Cell Adhesion Molecule-1
WB	Western Blot
WHO	World Health Organization
WY-14643	Peroxisome Proliferator-Activated Receptor-α agonist
